# Barriers to developing a valid rodent model of Alzheimer's disease: from behavioral analysis to etiological mechanisms

**DOI:** 10.3389/fnins.2015.00245

**Published:** 2015-07-29

**Authors:** Darryl C. Gidyk, Scott H. Deibel, Nancy S. Hong, Robert J. McDonald

**Affiliations:** Department of Neuroscience, Canadian Centre for Behavioural Neuroscience, University of LethbridgeLethbridge, AB, Canada

**Keywords:** Alzheimer's disease, rodent model, aging, hippocampus, memory, cognition, neurodegeneration, dementia

## Abstract

Sporadic Alzheimer's disease (AD) is the most prevalent form of age-related dementia. As such, great effort has been put forth to investigate the etiology, progression, and underlying mechanisms of the disease. Countless studies have been conducted, however, the details of this disease remain largely unknown. Rodent models provide opportunities to investigate certain aspects of AD that cannot be studied in humans. These animal models vary from study to study and have provided some insight, but no real advancements in the prevention or treatment of the disease. In this *Hypothesis and Theory* paper, we discuss what we perceive as barriers to impactful discovery in rodent AD research and we offer potential solutions for moving forward. Although no single model of AD is capable of providing the solution to the growing epidemic of the disease, we encourage a comprehensive approach that acknowledges the complex etiology of AD with the goal of enhancing the bidirectional translatability from bench to bedside and vice versa.

## Introduction

Alzheimer's disease (AD) is an insidious, devastating, aging-related disease that has debilitating effects on cognition. AD is truly a plight of aging, with ~10 and ~40% of people over the age of 65 and 85, respectively, being affected (Alz.org, 2014; Mormino, 2014). It is estimated that there are currently five million cases of AD in the United States and ~20–30 million cases world-wide (Lecanu and Papadopoulos, 2013; Prince et al., 2013; Iqbal et al., 2014). The number of AD cases is expected to double every 20 years, and triple by 2050 (Prince et al., 2013; Iqbal et al., 2014). An early-onset, familial form of Alzheimer's disease (FAD), which has been linked to mutations in the PS1 and PS2 genes has been identified. However, by far the most common form of Alzheimer's disease is the sporadic form (SAD), which accounts for ~85% of cases. Apolipoprotein ε4 allele (ApoE4), which is involved in amyloid beta (Aβ) trafficking and neural plasticity, is the strongest genetic precursor for SAD (Corder et al., 1993; Kalaria, 2000; Mahley and Rall, 2000; Kim et al., 2009; Fjell et al., 2014; for review see, Schellenberg and Montine, 2012). People carrying ApoE4 are two to three more times likely to develop SAD (Kalaria, 2000; for review see Kim et al., 2009). However, the presence of this allele is not causative. The current paper will focus mainly on SAD due to its much higher prevalence than FAD.

In addition to the toll that SAD takes on those afflicted and their families, this growing epidemic affects society as a whole. In people over the age of 60, it accounts for more disability years than cancer, stroke, cardiovascular diseases, and musculoskeletal disorders (Ferri et al., 2005). AD is a very costly disease, in 2014 it was estimated that the United States spent 214 billion dollars caring for AD patients, with the bulk of that money being spent on Medicare and Medicaid (Alz.org, 2014). Similar to the SAD prevalence statistics, the costs associated with this disease are expected to grow to 1.2 trillion dollars by 2050 in the United States (Alz.org, 2014). Developing better ways to diagnose, prevent, and treat SAD is of paramount importance for patients, their families, and society in general. Currently, pharmacological treatments for SAD are limited and their effects on memory are unimpressive (Mesulam, 2004), which is likely due to the complex etiology, heterogeneous pattern of pathology, and underlying mechanisms that continue to be elusive (McDonald, 2002).

SAD causes deleterious effects on various types of memory, such as: memory for personal experiences (episodic memory), spatial memory, and working memory (Tulving, 1993; Joseph et al., 2001; Serino et al., 2014). All of these types of memory rely heavily on intact hippocampus (HPC) and paraHPC function. Episodic and spatial memory are two of the first cognitive processes to be compromised, with executive function being affected in later stages of the disease (Grober et al., 2008; Serino et al., 2014). Neural degeneration, insoluble fibrous deposits in the form of amyloid plaques, and neurofibrillary changes such as Tau hyperphosphorylation are the key pathologies associated with SAD (Braak and Braak, 1991; Braak et al., 2011). Alzheimer's disease pathology progresses slowly and can take 10 years or more before AD is actually diagnosed (Jack et al., 2013). Medial temporal lobe areas involved in learning and memory such as, HPC, entorhinal (EC), and retrosplenial cortices are especially susceptible to these pathologies (Braak and Braak, 1991; for reviews see, Fjell et al., 2014; Small, 2014), as well as the effects of normal aging. In some cases in which only mild cognitive impairment is present, SAD can only be accurately diagnosed posthumously through *ex-vivo* examination (Snowdon et al., 1997). This suggests that the damage and degeneration caused by SAD pathology does not always result in cognitive deficits. Similarly, due to the slow progression and the heterogeneity of pathology in SAD, the pathology-behavioral threshold^*^ (^*^the point at which cognitive deficits can be detected due to brain pathology) itself is likely subject to a myriad of cofactors (McDonald, 2002). This threshold can vary from case to case, in a somewhat unpredictable manner. In addition to cognitive functioning, other co-morbidities such as, circadian rhythm dysfunction, depression, aggression, or psychosis can be associated with AD (Coogan et al., 2013; Lecanu and Papadopoulos, 2013).

As discussed in our previous work, we argue that the etiology of SAD is complex, and the pathology associated with SAD, as well as the behavioral consequences of the pathology are heterogeneous (McDonald, 2002; McDonald et al., 2010; Craig et al., 2011). One major issue is how the mechanisms of HPC dysfunction in SAD might be more diffuse and inconsistent across the entire system, which makes it difficult to detect behaviorally. This has been especially true in studies that use rodents. The gold standard behavioral tasks that have been employed with great success in the past to elucidate HPC-dependent behaviors in intact and HPC damaged rodents seem to fall short when attempting to reveal and quantify cognitive deficits in rodent models of SAD. Even in cases where there is considerable pathology and degeneration within the system, this does not always breach the behavioral threshold (Amtul et al., 2014a,b). We attribute this to the sparse, distributed nature of memory representations in HPC systems and the insensitivity of many of the classic HPC-dependent memory tasks to detect mild deficits in learning, memory and cognition. Additionally, it is our position that in some models, the induced or inbred SAD-like pathologies are often excessive, unifactorial, have unwanted side effects, and are not bidirectionally translatable. That is, as will be discussed below, not only are there difficulties in translation from bench to bedside, but from clinical cases to primary research as well. This issue, in combination with the difficulty in quantifying SAD-like behavioral impairments in rodents, represents a considerable impediment to advancements in the field of AD research. Although it may be tempting to seek out a universal rodent model of SAD, we maintain that the search for a single AD behavioral phenotype in rodents may be misguided due to the complexity of the disease.

The main goal of this paper is to address what we see as disconnect between the clinical reality of SAD and the primary rodent research in this field. Our hypothesis is that this disconnect is due to a reliance on inappropriate animal models and insensitive behavioral tasks. First, we will briefly discuss the evidence for the complexity and distributed nature of the HPC-based memory system and the behavioral tasks employed to identify the behavioral correlates of HPC dysfunction. Our focus on the HPC is based on our strongly supported assumption that the HPC is a major target of brain dysfunction in SAD. Second, we will evaluate some of the current rodent models of AD and identify what we perceive as barriers to progress in primary AD research. Lastly, we will synthesize these points of view and discuss them within the context of our cofactor theory of SAD. Our theory is that the cofactor model and more sensitive behavioral tasks will better represent aspects of human SAD that are typically absent in animal models, such as mechanism heterogeneity, AD sex differences, and nature of brain pathology. The issues raised in our paper speak to the topic of this special issue on how to progress in the fields of neuroprotection and rehabilitation by presenting important confounds associated with current rodent models of SAD and offering suggestions on how to move forward. Impactful progress in the fields of neuroprotection and rehabilitation, in our view, requires development of valid rodent models and behavioral tools for SAD that are specific for this devastating brain disease.

## The HPC and memory

In this section we will provide arguments and support for a simple idea that we feel has significant implications for research directed at developing rodent models of SAD. The idea is based on the fact that many of the well-accepted behavioral assays for hippocampal function were developed using techniques that render the HPC almost completely dysfunctional (complete lesions or inactivations). This is fundamental work but we believe that valid rodent models of SAD will actually produce partial or heterogeneous dysfunction of the HPC. This means that although significant portions of the HPC may be dysfunctional in a specific rodent model of SAD, standard assays of HPC function might not detect these changes leading the researcher to erroneously conclude that the model was inadequate. Below we will review key anatomical and functional organization of the HPC formation that makes it more difficult to detect functional impairments than other systems that are organized in a different way.

### HPC anatomical organization

Justifiably, the HPC and its associated regions are a major focus of study with respect to any disorder that affects learning and memory, specifically diseases of aging like SAD. An extensive body of theoretical and empirical work has amassed and provides strong evidence for the crucial role of HPC and paraHPC areas in learning and memory. Classically, the HPC proper was considered to be primarily a straight-forward circuit with unidirectional flow of information. Although the flow of information in HPC is largely unidirectional, HPC neuroanatomy lends itself to both serial and parallel processing with extensive reciprocal extrinsic connectivity, and intrinsic connectivity, which constitutes multiple parallel closed loops (Amaral and Lavenex, 2007). The HPC receives large amounts of input from all neocortical sensory areas via the EC and in turn, HPC efferents are sent back to the neocortex through the subiculum and EC (Amaral and Witter, 1989; Amaral and Lavenex, 2007). The HPC system is thought to form and maintain distinct multisensory associations, which are hypothesized to support unique, cohesive episodic memory representations (Sutherland and Rudy, 1989; Gruber and McDonald, 2012). These representations allow the organism to navigate through space effectively, temporally order events, and discriminate highly ambiguous situations using pattern separation/completion mechanisms (Morris et al., 1982; Sutherland et al., 1983; Leutgeb et al., 2004; Mankin et al., 2012; Hunsaker and Kesner, 2013; Serino et al., 2014).

The HPC and paraHPC regions generate and support allocentric (reliance on distal cues) and egocentric representations of the world (reliance on ones' position), which then can be integrated to form conjunctive units (Wilber et al., 2014). These representations can then guide future behavior, and are hypothesized to be one of the key substrates of memory. For example, spatial navigation can be mediated by distal allocentric, or egocentric idiothetic (self-movement generated) cues (O'Keefe and Nadel, 1978; Etienne et al., 1988; Maaswinkel et al., 1999; Martin and Wallace, 2007). Remarkably, rodents can use idiothetic cues in the absence of distal cues to navigate back to their starting location after foraging for food (Etienne et al., 1988; Maaswinkel et al., 1999; Martin and Wallace, 2007). This phenomenon is referred to as path integration and is dependent on the hippocampus (Maaswinkel et al., 1999) and the septohippocampal cholinergic system (Martin and Wallace, 2007).

The HPC system is able to create these allocentric and egocentric representations by encoding and processing signals from place cells, head-direction cells, and grid cells (Derdikman and Moser, 2010). In the rat, place cells generate an internal map of the environment, thereby providing an allocentric representation of locations within said environment (O'Keefe and Dostrovsky, 1971; McNaughton et al., 1983; O'Keefe and Speakman, 1987). These representations are stable over time and within each environment, but have the capability of rate and global remapping (Muller and Kubie, 1987; Leutgeb et al., 2005). Head direction cells are neurons that show high rates of firing when the animal's head points in a specific direction. These neuronal firing rates return to baseline when the subject's head turns about 45° from this preferred direction. Unlike place cells, these neurons are orientation specific, location invariant, and appear strongly dependent on the vestibular system. Head-direction cells are thought to contribute egocentric information about the direction of visual attention (Taube, 1998). Neurons classified as grid cells fire action potentials in small clusters with the clusters forming the vertices of a grid of equilateral triangles. Grid cells are found in the medial entorhinal cortex (MEC) and are posited to support the process of updating the rat's position within a spatial map via self-motion information, which is sometimes called path integration (Moser et al., 2008). In humans, and perhaps rodents, another paraHPC region, the retrosplenial cortex is thought to transform and integrate allocentric and egocentric representations generated in HPC regions (Maguire, 2001; Vann et al., 2009). Together, this network constructs and represents space in the brain. This multisensory construct can then be used to guide behavior and recall events associated with these places.

Another interesting feature of the HPC system is how it can represent seemingly endless amounts of information without experiencing catastrophic interference (McClelland et al., 1995). Evidence from computational and *in vivo* studies strongly suggests that memory representations are sparsely coded over distributed networks of neurons (Moser and Moser, 1998; Leutgeb et al., 2007; Wixted et al., 2014). This is hypothesized to greatly increase the associative capacity of HPC networks and lower the probability of interference between representations.

The complex, sparse, and distributed nature of memory representations in the HPC system becomes a considerable obstacle to overcome when attempting to study moderate, diffuse damage, characteristic of early to mid-stage SAD. What we refer to as the “gold standard” HPC-dependent behavioral tasks used to elucidate learning and memory deficits in rodents might be insensitive to this kind of incomplete hippocampal damage. Classic versions of the 8-arm radial arm maze (RAM; Olton et al., 1979), Morris water task (MWT; Morris et al., 1982; Sutherland et al., 1983), path integration task (Maaswinkel et al., 1999), and contextual fear conditioning (Sutherland and McDonald, 1990), have been useful in identifying HPC deficits on a behavioral level in the past. These behavioral tasks have the benefits of being well described and methodologically uncomplicated. However, as the field of HPC learning and memory research evolves, it is becoming clear that these “gold standard” tasks have limitations. These limitations become apparent when attempting to investigate HPC system damage or degeneration that is incomplete, diffuse, or heterogeneous. For example, in the hidden platform MWT, Moser et al. (1995) demonstrated that rats are able to learn and perform the task with ~74% of their HPC lesioned. Due to the extensive lesions, the authors referred to the small amount of dorsal HPC (dHPC) tissue as a “minislab.” Similarly, in single exposure contextual fear conditioning, rats given incomplete HPC damage after acquisition can display freezing behavior (Kim and Fanselow, 1992; Lehmann et al., 2007). These studies suggest that animals can display intact memory in these tasks despite substantial damage to the HPC. Again, we believe this can be attributed to the sparse and distributed nature of HPC memory representations. Additionally, altered experimental procedures such as repeated exposure, distributed training, or a within-subjects design involving multiple retention sessions instead of single exposure in contextual conditioning, can confound the HPC-dependent nature of the task (Lehmann et al., 2009). Engaging extrahippocampal memory systems renders the task ineffective for quantifying HPC-based memory impairments. Along this line, in a section below, anterograde and retrograde memory impairments will be discussed in the context of multiple memory systems.

### Multiple levels of functional organization of the HPC

Anatomical, pharmacological, and electrophysiological studies seem to suggest that the organization of the hippocampal formation follows certain topographical principles that could provide the substrate for memory-based behavioral subsystems. These include, but are probably not limited to, functional specificity at the intrahippocampal subfields, along the septotemporal axis, and various levels of anatomical organization between different portions of the EC and HPC.

#### Functional specificity at the HPC subfield level of description: dentate gyrus, CA3, and CA1

One obvious organizing principle of the functions of the HPC is the different subfields found within the structure. Briefly, extensive projections from the EC terminate on granule cells in the dentate gyrus. The granule cells in this subfield, in turn, project to the CA3 region via the mossy fiber system and synapse on pyramidal neurons there. CA3 pyramidal cells project to the CA1 region via the Schaffer collateral fiber system (Amaral and Witter, 1989).

There is evidence suggesting that each of these regions makes a specific contribution to hippocampal processing and functions. For example, it has been shown that the dentate gyrus and CA1 regions make differential contributions to spatial learning with dysfunction to the former impairing performance, while CA1 dysfunction enhances performance (Okada et al., 2003). Further, the dentate gyrus has been implicated in pattern separation, although this remains controversial (Spanswick and Sutherland, 2010). Others have recently shown that both the CA1 and CA3 contribute to the acquisition of context-dependent extinction but that CA1 is specifically required for contextual memory retrieval (Ji and Maren, 2008). Electrophysiological and lesion data suggests that the CA3 region is involved in rapid one-trial place learning, answering an unexpected question, pattern completion, and some forms of sequence learning (Zhou et al., 2012; Kesner and Rolls, 2015).

The implications of this regional specificity of function in the HPC are that SAD may preferentially compromise one or more of these systems and not the other, or damage all of these systems in one extent of the HPC but leave these systems intact in another part. In the former case, this may lead to more specific and subtle impairments that would require specific and sensitive assays for those subregional functions. In the latter case, memory impairments would be hard to detect unless one knew *a priori* which part was compromised and there was a behavioral assay available that was sensitive to the functions of that region.

#### Functional circuits via interactions between portions of EC and HPC: Medial and lateral EC and longitudinal strips

There are other ways to conceptualize information processing in HPC and associated intrinsic functional circuitry. For example, the major input to the HPC from all major cortical areas arises from the EC. Based on anatomical criteria, the EC has been subdivided into medial and lateral subregions (Witter et al., 2000). These subregions project into the HPC via different fiber tracts called the medial and lateral perforant path. With segregated input paths, different cortical inputs, and termination patterns in the different subregions of HPC it is reasonable to believe that these regions provide different information to the HPC and subserve different memory-based behavioral functions. Briefly, based on anatomical, electrophysiological, and behavioral evidence it has been argued that the MEC is preferentially involved in spatial navigation supported by idiothetic cues (self-motion) and the lateral EC processes local cues, cue configurations, and cue locations (Ferbinteanu et al., 1999; Knierim et al., 2014).

There has been a different way of conceptualizing HPC information processing presented based on other organizing anatomical principals of the HPC. For example, there are other interesting topographical relationships between the EC and HPC that have led us to alternative ways of conceptualizing information processing and potential functional subcircuits in the HPC. Briefly, perpendicular to the traditional medial/lateral EC division there is a second system of parallel circuits which separates the dorsal and ventral HPC and we have argued that this organization is the substrate for memory-based behavioral subsystems. The current stage of the model indicates at least two hypothetical memory-based behavioral subsystems within the EC-hippocampal formation. The DORSAL HIPPOCAMPAL CIRCUIT includes the lateral entorhinal cortex (LEC) strip, lateral nucleus accumbens, and the anterior prelimbic cortex. The VENTRAL HIPPOCAMPAL CIRCUIT includes the MEC strip, the medial nucleus accumbens, the basolateral amygdala, and the posterior prelimbic cortex.

#### Behavioral evidence for functional circuits in dorsal and ventral HPC

Our laboratory has collected a body of evidence to suggest that these different subsystems have different effects on memory-based behaviors.

##### Conditioned place preference (CPP)

The conditioned place preference task is an appetitive classical conditioning paradigm that uses distal spatial cues, like those used in place navigation tasks, as the conditioned stimuli. We showed that acquisition of this task requires a synergistic interaction between the amygdala-and hippocampal-based learning and memory systems (McDonald and White, 1995b; Ferbinteanu and McDonald, 2001) with the HPC providing complex spatial information and the amygdala providing access to reward information. Assessment of the functional significance of the topographical organization of the EC showed that: (1) rats with dorsal HPC lesions are impaired at acquisition of the same task suggesting that the dorsal hippocampal circuit is the crucial site for acquisition of this task (Ferbinteanu and McDonald, 2001); (2) rats with ventral hippocampal lesions or fornix lesions show enhanced acquisition on the CPP task suggesting that the ventral hippocampal circuit actively inhibits the amygdala-based learning and memory system (McDonald and White, 1995a; Ferbinteanu and McDonald, 2001).

##### Water task

The water maze procedure we use is a variant of the MWT and is a cue/place task (McDonald and White, 1994). The task consists of alternation of visible and invisible platform training. This procedure has various advantages over the traditional invisible platform water task (MWT). Using this task we have demonstrated that although the dorsal pole of the HPC is more efficient than the ventral pole in supporting place navigation, the dissociation is not absolute (Ferbinteanu and McDonald, 2000, 2003). This interpretation differs from that of Moser and colleagues (Moser et al., 1993; Moser and Moser, 1998). We have also reported that MEC input to the HPC but not LEC input is critical for efficient place learning in the water task (Ferbinteanu et al., 1999).

##### Discriminative fear conditioning to context

We have developed a powerful set of procedures for studying fear conditioning to context (Antoniadis and McDonald, 1999, 2000, 2001). Recently we have shown that both the amygdala and HPC are learning and memory systems that differentially participate in fear conditioning to context. Using this task we have demonstrated that active comparison (active place preference in which the animal is free to move back and forth between the paired and unpaired chamber via a connecting tunnel on the test day) of multiple cues with different emotional valence requires both dorsal and ventral HPC. Conversely, discriminative freezing which does not require active comparisons of cues was unaffected by either lesion (Ferbinteanu and McDonald, 2001). Complete neurotoxic lesions of the HPC disrupt both discriminative freezing and preference suggesting that these learning and memory functions require integration along the septo-temporal axis of the HPC. The behavioral evidence reviewed above provides evidence for the proposed memory subsystems in the HPC and extends our understanding of complex interactions between these HPC-based memory subsystems and other parts of the brain.

#### Demonstrations of differential functional effects of anterograde vs. retrograde HPC lesions

The asymmetry of anterograde and retrograde lesion effects on HPC-dependent behaviors provides another experimental consideration when employing classic HPC memory tasks to investigate behavioral impairments. In contextual fear conditioning, rats that receive HPC lesions prior to the single shock-context pairing display intact memory on retention tests (Maren et al., 1997; Wiltgen et al., 2006), even when HPC damage is extensive ~80% (Sparks, 2011). This could be due to the residual hippocampal tissue being capable of acquiring the representations required to learn the shock-context association. An alternative explanation, especially cases of severe or complete damage, is that some non-HPC memory system is capable of acquiring and supporting contextual fear memory in the absence of the HPC. This idea is supported by multiple memory systems theory (White and McDonald, 2002) and demonstrations of its proposed principals (McDonald and White, 1994). As mentioned above, the Moser group found that in MWT, rats with ~74% damage to the HPC could learn the location of the hidden platform. However, other studies have shown deficits in the anterograde direction with incomplete HPC lesions in MWT (Sutherland et al., 1983; Sutherland and Rudy, 1988; McDonald and White, 1994; Ferbinteanu et al., 1999). Although not straight-forward, it has been proposed that retrograde effects are more easily elicited than anterograde effects in HPC-dependent tasks (Morris, 2007). The somewhat elusive nature of retrograde impairments on HPC-dependent tasks in animals that have incomplete damage might be a function of the aforementioned distributed nature of the memory representations. Intact pattern separation/completion mechanisms are likely responsible for this in contextual fear conditioning, and could require less residual tissue to function adequately. Behavioral compensation or a switch of strategy could be responsible for the same anterograde/retrograde phenomenon in MWT. Another likely and perhaps complimentary explanation, is that the behavioral tasks employed might not require the animal to use effortful memory retrieval and the emotional salience of cues within the task (appetitive or aversive), or the task itself, provides additional stability to memories, which could serve to “unburden” more complex functions of the HPC (Gruber and McDonald, 2012).

### Summary

Acknowledging the complexity of HPC and paraHPC contributions to learning, memory, and cognition, as well as the interaction of these areas with others that support aspects of mnemonic functions may provide a key to improving the characterization of complex SAD pathology and associated behavioral correlates in rodent models of the disease. Careful consideration of all the experimental intricacies surrounding the visualization of HPC damage, pathology, and representative behaviors must be appreciated and taken into account when designing studies involving rodent models of SAD.

## Alzheimer's disease: Current approaches

The etiology of AD is very complicated. This is demonstrated by the fact that most individuals carrying ApoE4, and a subset of those with other biomarkers of AD never develop AD. For example, Schneider et al. (2007) observed that 24.2% of the brains from people in a retirement home with AD neuropathology were asymptomatic. This is likely because markers of AD, such as, amyloid plaques are not always positively correlated with degree of cognitive impairment (Giannakopoulos et al., 2003). Similarly, total HPC volume is not the best marker for AD because accelerated atrophy is a normal part of aging (Driscoll et al., 2009). Furthermore, there are other cognitive disorders, such as semantic dementia that have similar amounts of hippocampal atrophy (La Joie et al., 2013 for review see, Maruszak and Thuret, 2014).

Joseph et al. (2001) adopted a multi mechanistic approach by theorizing that membrane alterations, inflammation, and oxidative stress contribute to neurodegeneration and ultimately cognitive impairments. Along this line, it is currently believed that AD has a very complex etiology consisting of many risk factors (Joseph et al., 2001; McDonald et al., 2010; Craig et al., 2011). As highlighted by the statistics presented in the beginning of this section, by far the strongest predictor of developing AD is age (Kawas et al., 2000; Joseph et al., 2001; Fjell et al., 2014).

**Figure 1:**
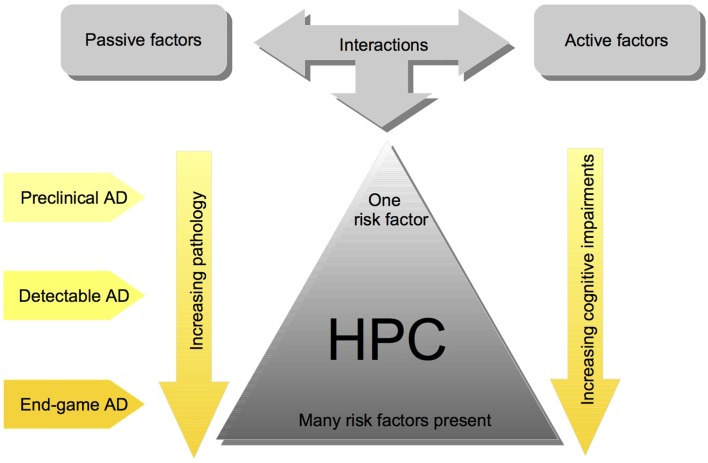
**Simplified schematic of the cofactor theory of SAD illustrating the interaction between risk factors, and their impact on HPC pathology and cognition**. Although these interactions are complex and the pathology is often heterogenous, the cofactor theory can be utilized to model different stages of Human SAD. Most importantly, the theory presents numerous, testable ideas that have yet to be assessed at all levels of analysis including: mechanisms of HPC dysfunction in preclinical animal models, clinical research assessing the potential for subgroups of individuals with different combinations of cofactors, and assessment of different preventative measures and treatment options.

We have proposed a cofactor approach to explain the complex etiology of SAD (McDonald, 2002; McDonald et al., 2010; Craig et al., 2011). Within this model, risk factors, which predominately affect brain areas that mediate learning and memory, have an additive effect, such that hippocampal pathology worsens with more risk factors (see Figure 1). There are *active factors*, such as stroke or seizure, which cause neuronal death, and *passive factors*, such as cholinergic depletions or Aβ, which make the brain more susceptible to the damaging factors (for review see, McDonald, 2002). The idea is that there would be very little or no brain pathology and cognitive impairment when one of these factors is present, but several of these factors would create more severe brain pathology and cognitive impairments (see Figure 1). The cofactor model suggests that effects of AD treatments are heterogeneous despite patients having very similar symptomology because it is likely that across patients different mechanisms are elicited by the specific combination of risk factors present (McDonald et al., 2010).

Generally, risk factors are believed to affect hippocampal functioning primarily via neurodegeneration or altering plasticity/signal transduction in the HPC (for reviews see, Joseph et al., 2001; McDonald et al., 2010; Fjell et al., 2014). For example, functions and/or molecules necessary for hippocampal dependent memory, such as neurogenesis, acetylcholine, and brain derived neurotrophic factor are all altered in AD patients (Honea et al., 2013; Lazarov and Marr, 2013; Maruszak et al., 2013; for reviews see, Craig et al., 2011; Maruszak and Thuret, 2014). Acetylcholine particularly has been linked to AD, as AD patients have a loss of cholinergic neurons in the basal forebrain (Davies and Maloney, 1976; Mesulam, 2004; Craig et al., 2011). Cholinergic depletion of the medial septum dramatically decreases the amount of acetylcholine in the hippocampus and in animal models can impair memory in certain tasks, such as those that assess spatial working memory or path integration (Lehmann et al., 2003; Martin and Wallace, 2007; Craig et al., 2011), but typically not declarative spatial memory (Berger-Sweeney et al., 1994; Baxter et al., 1996). Although it is unclear if cholinergic depletion precipitates AD or is a symptom, interestingly, acetylcholine agonists are some the most common and effective drugs used to ameliorate memory impairments in AD patients (Craig et al., 2011). However, it should be noted that the beneficial effects of acetylcholine agonists are small and often not long-lasting (Craig et al., 2011). Our theory is that cholinergic depletion increases the likelihood of AD by making the hippocampus more prone to damage or dysfunction when presented with other risk factors (Craig et al., 2011).

### Animal models of Alzheimer's disease

As a result of the complex and largely unknown etiology of AD, animal models are particularly useful and ultimately necessary to study possible causes and treatments of this disease (Sodhi et al., 2014). Not surprisingly, due to the contention surrounding the etiology of AD, there is no standard animal model of AD (Joseph et al., 2001; Lecanu and Papadopoulos, 2013; Iqbal et al., 2014; Sodhi et al., 2014). Animal models attempt to create AD like brains and cognition by changing the genome, or by adding chemicals to the brain (Sodhi et al., 2014). We will discuss the various strengths and weaknesses of some of the animal models of AD.

#### Transgenic animal models of AD

Upon the discovery that mutations in specific genes cause familial AD, transgenic mice with similar genomes were created. Transgenic mouse and rat models of AD are currently the most popular animal model of AD (Lecanu and Papadopoulos, 2013; Iqbal et al., 2014; Sodhi et al., 2014). Most of the transgenic models involve genetic manipulations that lead to over expression of Aβ and/or promote the formation of neurofibrillary tangles (for reviews see, Braidy et al., 2012; Sodhi et al., 2014). While there are many transgenic models, typically those that pair several mutations have been the most successful at mimicking AD pathology (Braidy et al., 2012; Lecanu and Papadopoulos, 2013; Sodhi et al., 2014). Some examples include models, in which amyloid precursor protein (APP) mutations have been paired with PSEN1 (*APP*/*PS1*), and, or tau mutations (*APP*/*tau; APP/PS1/tau*) (Borchelt et al., 1997; Lewis et al., 2001; Oddo et al., 2003). Depending on the specific mutations used, these models elicit memory impairments that are thought to be due to increased Aβ and/or neurofibrillary tangles (for reviews see, Braidy et al., 2012; Sodhi et al., 2014).

##### Evaluation of transgenic models

The primary strength of transgenic animal models is that they closely resemble FAD, in terms of the mechanism mediating AD pathology. The use of transgenic models has been crucial, as they have elucidated a lot of the molecular mechanisms/biomarkers that contribute to AD pathology (Balducci and Forloni, 2011; Braidy et al., 2012; Lecanu and Papadopoulos, 2013). That being said, transgenic animal models of AD possess weaknesses, some of which we feel are hard to overlook. First, these models only represent FAD, which as mentioned above, only accounts for ~5% or less of all AD cases (Balducci and Forloni, 2011; Lecanu and Papadopoulos, 2013; Iqbal et al., 2014).

Second, many of these models produce pathologies that might not be representative of human AD pathology. Many transgenic models overexpress APP and thus these models are producing phenotypes that are not just a result of increased Aβ loads (Joseph et al., 2001; Saito et al., 2014). For example, in some transgenic models amyloid pathology is created by the unrealistic overexpression of APP (5–10 fold) and then subsequent Aβ levels (5–12 fold) (for review see, (Balducci and Forloni, 2011)). These models are similar to very severe cases of dementia, which report up to an 11.9 fold increase of Aβ_*x*−42_ in the temporal lobe (Näslund et al., 2000). However, this varies greatly depending on the brain region studied, as there was only a 4.6 fold increase of Aβ_*x*−42_ in the frontal cortex of the same patients (Näslund et al., 2000). Similarly, studies using patients with early dementia or a sample of sporadic AD patients, report as little as ~1.5 fold increase in Aβ depending on the Aβ fragment and brain region (Näslund et al., 2000; Klunk et al., 2004; Li et al., 2004). Additionally, in many transgenic models oligomeric Aβ expression is also very different from that found in human AD patients (for review see, Balducci and Forloni, 2011). Thus, Balducci and Forloni (2011) stated that in contrast to humans, the Aβ pathology created by transgenic models is excessive and is more similar to the rare FAD.

In addition to unrealistic Aβ overexpression, many of the transgenic models that use tau mutations produce unrealistic tau pathology. First, tau mutations have never been found in AD patients (Tackenberg and Brandt, 2009; Umeda et al., 2014). Second, some of the FTDP-17 tau mutation models produce tau that is more toxic than wild type tau and the mechanisms for how Aβ interacts with tau is even different from wild type tau in these mutants (Tackenberg and Brandt, 2009). Furthermore, some of these models produce neurofibrillary tangles in the absence of Aβ, which contradicts the classic theory that Aβ precipitates abnormal tau pathology (Hardy and Selkoe, 2002; Frank et al., 2008; Umeda et al., 2014).

There are other less severe weaknesses of transgenic models. For example, these transgenic models typically do not cause very much neurodegeneration, which some consider to be the hallmark of AD pathology (Lecanu and Papadopoulos, 2013). Another flaw is the timing of the presentation of brain pathology and behavioral impairments. For example, typically these animals possess the genetic mutations from birth, so there is not always much disease progression (Sodhi et al., 2014). However, behavioral impairments in transgenic mice models are not always seen until the animals have aged, which makes it hard to determine exactly how the induced AD pathology is affecting the brain and behavior mechanistically because aging also affects many other processes (Joseph et al., 2001).

Additionally, for several reasons, it is very hard to compare transgenic models of AD. First, many different mice strains or hybrid strains are used for transgenic mouse models (Joseph et al., 2001). This strain heterogeneity makes it hard to compare transgenic models, as there are strain specific differences in the performance of behavioral tasks (Joseph et al., 2001). Hybrid mouse strains can also have vision problems that confound any results obtained from behavioral testing (Borchelt, 1998; for reviews see, Joseph et al., 2001; Brown, 2007). Second, aspects of pathology, such as the location of Aβ plaques and neurofibrillary tangles, vary depending on the promoter region used to incorporate the transgene into the animal's genome (Braidy et al., 2012; Lecanu and Papadopoulos, 2013). Therefore, different models using similar genetic mutations can produce very different brain pathologies and cognitive deficits.

Finally, the overwhelming majority of transgenic models use mice (Sodhi et al., 2014). This can be problematic because in contrast to the rat, less is known about the effect of lesions and pharmaceuticals on mouse behavior (Young et al., 2013). Furthermore, behavior in the “gold standard” hippocampal dependent memory tasks is not always analogous between species, as mice perform worse than rats in the MWT (Whishaw and Tomie, 1996).

#### Cofactor models

As discussed by Sodhi et al. (2014) many chemicals, such as exogenous Aβ, neurotoxins that target specific cell types, heavy metals, and sodium azide can be given to rodents to model AD. 192 IgG-Saporin is a commonly used neurotoxin that is favored by our and other labs because of its selective specificity for cholinergic neurons. We use 192 IgG-Saporin to reduce ACh in the hippocampus (>60%) by abolishing cholinergic neurons in the medial septum (Craig et al., 2011). Many neurotoxins produce cognitive impairments and aspects of AD pathology (for review see, Sodhi et al., 2014). However, these models typically only produce partial AD pathology because primarily one factor is used. For several reasons, this is not likely representative of true AD pathology. First, multiple factors are involved, and second, as mentioned many of these studies use unrealistic factor amounts (Joseph et al., 2001). While, this approach is beneficial because it helps verify whether or not a factor is a potential mechanism, this approach does not create realistic AD pathology.

Our lab and other labs utilize a cofactor animal model that involves the presentation of various combinations of risk factors to rodents. We use a sub-threshold dosing strategy, so the idea is that one risk factor will have a minimal effect on brain pathology and cognition, however, multiple factors presented concomitantly will exacerbate brain pathology and produce cognitive impairments. To our knowledge, Dornan et al. (1993) provided the first evidence of the cofactor effect by only observing impaired MWT acquisition when both Aβ_25−35_ and ibotenic acid were injected into the HPC and not when each factor was presented in isolation.

We have presented various combinations of risk factors to rats (for review see, Craig et al., 2011). As indicated above, age is the strongest risk factor for AD. However, it is difficult to determine why aging induces AD as many brain pathologies occur during aging (Joseph et al., 2001; Fjell et al., 2014). Thus, we try to determine which of the age-induced pathologies contribute to AD by creating these pathologies in young adult male Long Evans rats.

**Figure 2:**
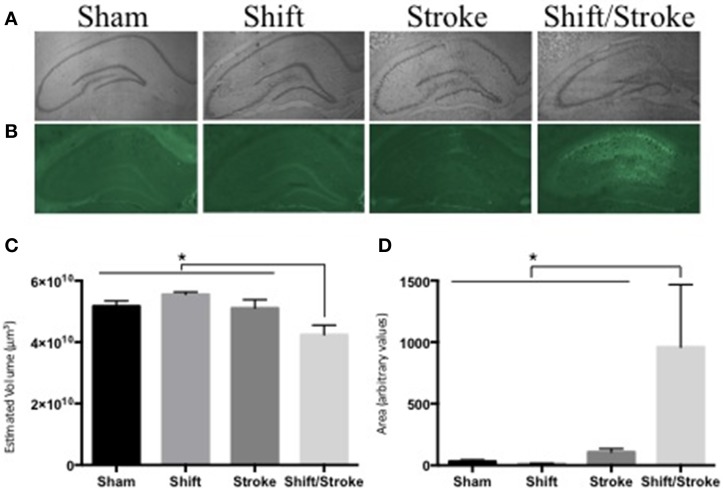
**Hippocampal volume and fluoro Jade staining in rats with mini-hippocampal strokes and acute circadian rhythm disruption**. Rats received: incisions and sutures (sham); 6 days of photoperiod shifting (shift; Devan et al., 2001; Zelinski et al., 2014); 0.5 μl of 6 pmol endothelin-1 (ET-1) was injected into the hippocampus via two sites bilaterally (stroke; McDonald et al., 2008); 6 days of photoperiod shifting and then ET-1 infusions (shift/stroke). Twenty-two days after these manipulations, the rats were perfused transcardially with PBS and 4% buffered paraformaldehyde. Sliced sections (40 μm) were either stained with Cresyl violet for volume analysis, or 0.0004% Fluorojade-B (FJ) for identification of damaged neurons (McDonald et al., 2008). Whole hippocampal volumes were measured using the Cavalieri method via StereoInvestigator (Microbrightfield, Williston, VT). Every sixth section was counted with an average of 14 sections assessed for each animal. If sections were missing from a series (on average 1 section per animal), stereo investigators correction for missing sections was used. For the FJ analyses, ImageJ was used to create a threshold that selected the FJ fluorescent signal in the whole hippocampus and then the area of this selected region was quantified in each of three representative sections per animal (−2.56, −3.8, and −4.8 mm from bregma respectively). **(A)** Representative images used for the volume analyses: sham (*n* = 8); shift (*n* = 9); stroke (*n* = 8); shift/stroke (*n* = 6). **(B)** Representative images used for the FJ analyses: sham (*n* = 5); shift (*n* = 7); stroke (*n* = 8); shift/stroke (*n* = 6). **(C)** A One-Way ANOVA indicated that hippocampal volume differed among the groups [*F*_(3, 27)_ = 6.058, *p* = 0.003] and a planned comparison indicated that the shift/stroke had smaller volumes when compared to the other groups (*p* = 0.001). **(D)** A natural log transformation was performed as Levene's test was violated and a One-Way ANOVA revealed that the average area per section of FJ signal differed between the groups [*F*_(3, 22)_ = 8.918, *p* < 0.001]. A planned comparison indicated that the shift/stroke group had a greater area of damaged neurons when compared to the other groups (*p* = 0.001).

We have demonstrated that the following pairings of active and passive factors have affected hippocampal dependent behavior and/or pathology: cholinergic depletions + stroke (Craig et al., 2009a); cholinergic depletions + kainic acid seizures (Craig et al., 2008b); aging + hippocampal mini-strokes (Driscoll et al., 2008); stress + hippocampal mini-strokes (McDonald et al., 2008). Recently, as predicted by our theory, for the first time, we observed that the pairing of two active factors—acute circadian rhythm disruption and hippocampal mini-strokes—reduced total hippocampal volume and increased neurodegeneration in the HPC (see Figure 2). This finding is notable because as will be discussed below factor combinations do not always exacerbate brain pathology, even in some instances when they elicit behavioral impairments.

Interestingly, some active and passive factor pairings have caused little or no behavioral impairments: circadian rhythm disruption + cholinergic depletions (Craig et al., 2009b); Aβ + striatal mini-strokes (Amtul et al., 2014a,b; Keeley et al., 2015). Similarly, the pairing of circadian rhythm disruption + cholinergic depletions did not cause hippocampal pathology (Craig et al., 2009b). Surprisingly, we have also observed varying degrees of behavioral impairments and hippocampal pathology when two passive factors were paired together: stress + cholinergic depletions (Craig et al., 2008a); stress + Aβ (Deibel et al., unpublished); cholinergic depletions + Aβ (Deibel et al., unpublished). These data sets suggest that the cofactor theory is not a basic two-hit theory, nor are the active and passive requirements always met. For example, the shift/stroke data presented above, particularly demonstrates that the co-factor theory is not just a two-hit theory and depends on the specific factors used, as circadian rhythm disruption had no effect on pathology and behavior when paired with cholinergic depletions (Craig et al., 2009b). With this in mind, recently we have argued that some of these factors have both passive and active components. For example, the finding that stress exacerbates pathology when paired with a passive factor (cholinergic depletions; Aβ) or active factor (mini-stroke) suggests that it could have both active and passive characteristics (Deibel et al., unpublished; McDonald et al., 2008). Similarly, Aβ likely has both passive and active characteristics, as some pathology is more severe when Aβ is paired with a passive (stress) or active factor (mini-stroke) (Amtul et al., 2014a,b; Deibel et al., unpublished).

In summary, as discussed in Craig et al. (2011), several patterns of results emerge from our data. Some factor combinations can elicit significant hippocampal neuronal cell death and hippocampal dependent learning impairments (shift/stroke data presented above; Driscoll et al., 2008; McDonald et al., 2008). Conversely, some factor combinations can result in no hippocampal pathology, but behavioral impairments in hippocampal dependent learning tasks (Craig et al., 2008a,b, 2009a,b; Keeley et al., 2015; Deibel et al., unpublished). Other factor combinations can cause hippocampal or striatal pathology, but no or very minimal behavioral impairments (Amtul et al., 2014a,b; Deibel et al., unpublished). Finally, factor combinations can have no effect on hippocampal pathology or behavior (Craig et al., 2009b). As a whole our data support the notion that AD pathogenesis is heterogeneous because hippocampal pathology/behavior depends on the specific combination of factors used.

Other labs obtained similar results with some of the risk factor combinations we have used: Aβ+ stroke (Tong et al., 2005; Whitehead et al., 2005a,b; Iwasaki et al., 2006; Whitehead et al., 2007); Aβ+ chronic hypoperfusion (Choi et al., 2011); cholinergic depletions + seizures (Jouvenceau et al., 1997; Silveira et al., 2000); Aβ_1−40_ dorsal hippocampal injections + stress (Huang et al., 2010); transgenic mice overexpressing APP + cholinergic depletions (Gil-Bea et al., 2012; Ramos-Rodriguez et al., 2013).

Other risk factor combinations have also been used: stroke + diabetes (Stewart and Liolitsa, 1999); stroke + stress (Madrigal et al., 2003); stress + seizures (Srinivasan et al., 2006); stress + aging (Lupien et al., 1998; Sapolsky, 1999). Finally, an interesting rat model produces all of the hallmarks of AD pathology, such as hippocampal dependent memory impairments, amyloid plaque deposits, hyperphosphorylated tau protein, and neurofibrillary tangles via chronic injections of a solution consisting of Aβ_1−42_, and the pro-oxidative inducing agents: ferrous sulfate and buthionine sulfoximine (Lecanu and Papadopoulos, 2013).

##### Evaluation of cofactor models

The biggest strength of the cofactor model is that, unlike transgenic models, it allows the etiology of SAD to be investigated via the presentation of various risk factors. There are three different approaches for presenting risk factors to rodents. As mentioned above, most cofactor models present risk factors to young adult animals. There are both positives and negatives to this approach. Presenting risk factors to young adult animals controls for age and thus allows a better assessment of risk factors and how they might interact to produce brain dysfunction and memory impairments. However, in reality these risk factors are occurring in an aged brain. Alternatively, risk factors can be presented to aged animals. Data from our and other labs suggest that pathology and cognition is exacerbated when risk factors are presented to older animals (Driscoll et al., 2008; Ramos-Rodriguez et al., 2013; Gidyk et al., unpublished). Finally risk factors can be presented to young or middle aged animals and then the animals are allowed to age before pathology and cognition are assessed. For example, Ramos-Rodriguez et al. (2013) found that transgenic mice over expressing APP were more impaired in hippocampal dependent tasks when they were allowed to age after receiving cholinergic depletions. We anticipate that in our model, this approach would vastly exacerbate hippocampal pathology and behavioral impairments. Although the last two approaches incorporate the aging risk factor, as previously mentioned it is hard to evaluate the contribution of other risk factors because aging is intermingled with these factors. For example, the visual acuity of rodents decreases with age (Brown, 2007), and thus could produce behavioral impairments that are perceived to be due to other risk factors. Thus, we believe that both young and aged animals should be used in concert when evaluating risk factors.

As with the transgenic models, within a species, different strains and exclusively males (will be discussed in detail below) are often used in cofactor models. Strain differences are well documented in learning and memory behavioral paradigms (Tinius et al., 1989; Andrews et al., 1995; Paré, 1996; Pryce et al., 1999; Cain et al., 2004; van der Staay et al., 2009; Thorpe et al., 2012). This is a major problem because across studies, it is unclear if behavioral differences are a result of the factors used or the strain or sex of species used. We and others feel that Long Evans rats are an ideal subject for an AD model because of their superior spatial learning abilities and the fact that their behavior and physiology have been extremely well documented (Lecanu and Papadopoulos, 2013).

The pathology induced by cofactors is going to vary depending on which cofactors are used. Thus, some cofactors will likely not elicit all of the hallmarks of AD pathology. While this could be considered a weakness of the model, we feel that this is a strength because SAD pathology varies greatly across people. On this view, the actual mechanism mediating the cognitive impairments might not be one of the classic hallmarks of AD pathology. In other cases, the other factors might exacerbate the effects of classic AD pathology. However, in both cases the cofactors play a key role in hippocampal dysfunction and related memory impairments.

#### Unjust criticisms directed at the cofactor model of SAD

Although there are a variety of perceived weaknesses for cofactor animal models of SAD, some of these criticisms might be unjust. This section will offer some alternative interpretations for some of these perceived weaknesses.

The most used criticism for animal models of AD is that these models fail to induce the amount of neurodegeneration seen in AD patients. There are a variety of reasons why we do not view this as a fault. First, at the first sign of altered cognition, AD pathology can take 10 or even 20 years to produce the post-mortem pathology of AD patients (Jack et al., 2010; Villemagne et al., 2013). In fact, neurodegeneration is not always the best marker for identifying AD (Driscoll et al., 2009; La Joie et al., 2013). Similarly, in rodents, lesion size does not always correlate with severity of behavioral deficits, especially in the motor domain (Metz et al., 2005; Alaverdashvili et al., 2008). As demonstrated by some of our cofactor combinations, and other studies measuring the effect of various brain insults on behavior, it is possible to have behavioral impairments without gross hippocampal damage (Jaspers et al., 1990; Wahl et al., 1992; Yamaguchi et al., 1995; Craig et al., 2008a,b, 2009a,b; Keeley et al., 2015; Deibel et al., unpublished). It is likely that altered synaptic plasticity is responsible for the impaired cognition that occurs early on in the progression of AD (Selkoe, 2002). It is possible that animal models which do not produce neurodegeneration might represent early AD pathology. Some suggest that a cure for AD is not possible because the primary mechanism is aging and it is going to be very difficult to replace dead or dying brain tissue (de la Torre, 2012). Instead, prevention is likely the best and only way to manage AD (de la Torre, 2012). Thus, the early stages of AD should be the focus of human clinical work and animal models for AD (Selkoe, 2002). Our theory is that cofactor models of SAD are more representative of human SAD than transgenic models of AD (see Table 1). As mentioned earlier, most of the transgenic AD models are more representative of extreme end game AD pathology. By logical extension, animal models of AD without neurodegeneration and minimal Aβ are arguably more important than a model with massive amounts of neurodegeneration or Aβ because they represent early AD.

**Table 1:** **Summarization of the hallmarks of human SAD pathology and how well transgenic and cofactor models represent these pathologies**.

**SAD pathology**	**Humans**	**Transgenic models**	**Cofactor models**
Aβ	~1–12 fold increase	~5–12 fold increase	~2.5 fold increase in several instances
Tau/Neurofibrillary tangles	Present, but not due to mutations	Can be present, but typically elicited by mutations	Can be present without mutations depending on the risk factors used
Neurodegeneration/Hippocampal atrophy	~15–20% reduction in hippocampal volume in mild AD	Can be Similar to humans but depends on the specific mutation and area of the hippocampus	Decreased hippocampal volumes (see Figure 2) or increased cell death depending on the risk factors used
Cognitive impairments	Episodic and working memory impairments	Typically impairments in the gold standard tasks depending on the mutations used, but mice behavior poses a potential caveat	Rats typically display subtle impairments in more sensitive variants of the gold standard tasks
Disease progression	10–20 years for end-game AD pathology	Some disease progression, but aging can't be differentiated from other mechanisms	Some disease progression, which can be precisely manipulated by experimenter

In addition to animal models being discounted because of minimal neurodegeneration, those that produce minimal cognitive impairments are also criticized. Contrary to this, Joseph et al. (2001) stated that some animal models were actually limited because the cognitive impairments were global and therefore did not resemble those experienced by AD patients. As will be discussed in the next section, the task must be taken into consideration when interpreting behavior in animal models of disease (Hånell and Marklund, 2014). Some of the tasks used to assess memory in animal models of AD are very resistant to the effects of damaging factors. For example, we recently observed that Aβ paired with either stress or cholinergic depletions elicited minor behavioral impairments during acquisition of the MWT (Deibel et al., unpublished). Along this line, rats can acquire and retain a spatial memory in the MWT with massive hippocampal lesions (Moser et al., 1995), therefore, even a minimal impairment in some of these tasks should not be overlooked.

### Strategies to detect incomplete damage to the HPC that should be utilized in animal models of SAD

As stated in the introduction, our theory is that many of the wrong behavioral tasks are being used and we hypothesis that more sensitive tasks would either detect memory impairments that were not detected by the “gold standard” tasks, or provide information as to which specific hippocampal sub-circuits are affected. Based on our experience, we believe there are several strategies to help deal with the problem of detecting heterogenous and/or incomplete hippocampal damage in animal models of SAD. One approach that has worked in our hands is designing learning and memory tasks that place a higher demand on hippocampal processing compared to standard versions of these (McDonald et al., 2004). For example, we have developed spatial (McDonald and White, 1995a), contextual (Frankland et al., 1998; Antoniadis and McDonald, 1999, 2000) and configural tasks (McDonald et al., 1997) that are highly sensitive, or not sensitive at all to HPC damage. The methodological strategy we used in these different experiments was to manipulate the level of cue ambiguity or overlap. Spatial, contextual, or configural tasks with high cue ambiguity were more sensitive to HPC damage than versions with low cue ambiguity. For example, with one cofactor combination, we observed normal acquisition in the standard MWT, but impairments in both a mass training session to a novel platform location and relearning of the original platform location (Craig et al., 2008a). These manipulations assess the ability of the hippocampus to rapidly form a novel representation in a familiar context.

A second strategy for detecting partial HPC dysfunction would be to use learning and memory tasks that likely require multiple functions of the HPC mediated by different subcircuits. For example, identifying whether or not a familiar object is out of context rather than the identification of a novel object, has been shown to be sensitive to incomplete damage to the HPC that is isolated to one subfield: the dentate gyrus (Spanswick and Sutherland, 2010). This task might be particularly sensitive to partial HPC damage because it might require both the medial and lateral EC contributions to HPC function (place and objects). Similarly, the Guzowski group has also demonstrated that rats given a drug that elicits neuroinflammation in the dentate gyrus, were unimpaired in the classic hippocampal dependent tasks, such as MWT, contextual fear conditioning, and novel object recognition, however, they were impaired in aversive and appetitive context discrimination tasks that are thought to require pattern separation (Czerniawski and Guzowski, 2014; Czerniawski et al., 2015).

Finally, a strategy used by our research group is based on the idea that there are multiple learning and memory systems in the mammalian brain and that they acquire and store different types of information during a particular experience. These systems are thought to interact either synergistically or competitively to control behavior (White and McDonald, 2002). Learning and memory tasks which induce competitions between learning and memory systems are important tools for detecting partial dysfunction of a particular system because competition reveals weakness. For example, we developed a competition test between stimulus-response vs. spatial representational control using a modified version of the cue/place task (Sutherland and Rudy, 1988). Briefly, rats are trained to swim to a cued platform in a circular pool located in the same spatial position for several days. On every 4th day, the cued platform is removed and a hidden platform is put in the same spatial location and the rats are trained to swim to that spatial position to escape. This pattern of training is repeated thrice for a total of 12 training days. On day 13, the visible platform is moved to a different spatial position and the subjects are allowed to swim to either the previously correct spatial position or the cued position. Rats with damage to the dorso-lateral striatum, a neural system thought to be important for stimulus-response learning, preferentially swam to the previously correct spatial location. Rats with damage to the HPC system preferentially swam to the cued position. Interestingly, the HPC damage was only partial and yet a powerful bias to the other learning and memory system was produced (McDonald and White, 1994).

An example of the utility of this type of competition task for applied approaches has also been demonstrated in several studies from our lab. Using an animal model of moderate exposure to alcohol during prenatal development, researchers showed that neurobiological mechanisms implicated in plasticity underlying learning and memory functions in HPC were compromised but no learning and memory impairments were revealed using the standard, hidden platform version of the water task. However, using the cue/place competition task, a HPC deficit was detected in the adult rats pre-exposed to low levels of alcohol during prenatal development. A similar HPC impairment was revealed using a one-trial place learning task that places a high demand on HPC circuitry because the subjects must learn new spatial positions in the pool each day to find the escape platform (Sutherland et al., 2000). This pattern of behavioral effects was found, in our view, because the low level exposure to alcohol during prenatal development induced partial HPC dysfunction and was only detected using more sensitive learning and memory tasks.

As a whole, the data presented in this section demonstrate that the behavior of animals with various brain pathologies can differ even when similar tasks are used that are thought to assess the same neural systems. These data sets also indicate tasks thought to rely on HPC circuitry vary on their level of sensitivity to complete vs. partial damage.

#### Wait a minute: many current rodent models of AD demonstrate severe learning and memory deficits

We suspect that most researchers involved in developing and utilizing rodent models of AD would not recognize the issue concerning rodent models of SAD producing partial HPC damage and the need for sophisticated strategies for detecting this type of dysfunction as a significant problem. The reasons for this are obvious. As previously described, most rodent models of AD are for the familial version and these models have tended to produce severe functional deficits. However, we feel that as the field progresses the models will more accurately reflect the conditions found in SAD patients and deficits will not be so global.

#### The use of episodic memory tasks in animal models of AD

In addition to the use of variants of the “gold standard” tasks, some suggest that other behavioral tasks might be more representative of the type of memory affected in human AD patients. For example, many drugs that improve memory or cognition in preclinical testing are often not successful in human clinical trials (Kimmelman and London, 2011; Crystal and Glanzman, 2013). Some argue that the wrong behavioral tasks are used (Joseph et al., 2001; Crystal, 2013; Crystal and Glanzman, 2013). It is possible that disconnect between animal models and human clinical trials exists because in animal models we often are not assessing the type of memory that might be effected in human AD patients.

As discussed in the introduction, episodic memories are more sensitive to the effects of aging and dementia related diseases than semantic memories (Hodges and Patterson, 1995; Perry et al., 2000; for review see Tulving and Markowitsch, 1998). Although spatial learning is hippocampal dependent (Morris et al., 1982; Sutherland et al., 1983), there are crucial differences between spatial and episodic memory. Primarily, some argue that after extensive training, spatial learning tasks can involve semantic memory (Crystal, 2013). Furthermore, spatial tasks involve allocentric spatial representations, whereas episodic memory tasks often involve egocentric representations (Davis et al., 2013a,b). Aging/brain pathology can differentially affect the processes mediating allocentric and egocentric representations (Davis et al., 2013a,b; Serino et al., 2014). Along these lines, it would interesting to assess path integration in animal models of AD. As path integration does not involve the use of allocentric cues it is possible that the AD like animals might be differentially impaired in path integration tasks compared to other spatial navigation tasks.

Although there is much debate about whether animals are capable of episodic memory, there are episodic like rodent memory tasks, which involve the discrimination of an event at a certain time or place (for reviews see, Crystal, 2013; Easton and Eacott, 2013). These tasks primarily differ from spatial learning tasks in that they have a temporal component and often involve less training than spatial learning tasks. Eacott and colleagues have used an episodic like memory task to evaluate behavioral impairments in a transgenic AD mouse model (Davis et al., 2013a,b). They demonstrated that normal aged and transgenic AD mice were impaired in the episodic like what-where-which task, but not in several other hippocampal dependent tasks: a temporal order task and what-where-when task (Davis et al., 2013a,b). We predict that this effect might be producible for some cofactor combinations. In fact, along with other criteria, this effect could be used to determine which co-factors or SAD models truly represent the SAD phenotype.

However, it should be noted that there are alternative explanations for disconnect between clinical studies and animal models. For example, the most likely explanation is that animal models artificially elevate levels of various AD pathologies and then treatments are used that target these pathologies specifically. However, as mentioned before, human AD pathology is so heterogeneous and hard to diagnose while alive, that a drug that mitigates a specific AD pathology in an animal model would likely only be effective in a very specific subset of AD patients.

#### Other factors that need to be considered in future models

In addition to the type of animal model and task, there are several other factors that need to be incorporated into future models. First, to our knowledge very few studies differentiate hippocampal pathology in the ventral and dorsal regions. As posited by Maruszak and Thuret (2014), the dorsal HPC (posterior HPC in humans) is required for spatial and temporal tasks that require fine contextual information, whereas the ventral HPC (anterior HPC in humans) is thought to be involved in representing general contextual information that is required for contextual discrimination (Ruediger et al., 2012). The finding in rats that the dorsal HPC is required for the standard version of the MWT and the ventral HPC is not, supports this notion (Moser et al., 1995). Interestingly, as discussed by Maruszak and Thuret (2014), in human AD patients, there is more neurodegeneration in the ventral compared to the dorsal HPC (Raji et al., 2009).

With this in mind, we predict that animal models of AD should have more severe pathology in the ventral hippocampus and be more impaired on tasks that specifically assess ventral hippocampal function. Surprisingly, some transgenic models find more pathology in the dorsal HPC (Rodríguez et al., 2008; Fuster-Matanzo et al., 2011). In contrast to the transgenic models, some of our recent cofactor combinations in rats appear to mirror the human pathology, by suggesting that the ventral HPC is more susceptible to risk factors. For example, just as in mice with ventral HPC lesions (Ruediger et al., 2012), we recently have observed that several passive factor combinations (stress + Aβ; cholinergic depletions + Aβ) only produced impairments early on in MWT acquisition (Deibel et al., unpublished). These data fit with the finding that the ventral HPC is more susceptible to the malaffects of mild chronic stress (Jayatissa et al., 2008) and chronic alcohol consumption (Lescaudron and Verna, 1985). In summary, more animal models of AD need to quantify hippocampal pathology in the dorsal and ventral regions, as these regions have different functions and in humans can be differentially affected during AD. Similar to performance in episodic memory tasks, this analysis might help undercover animal models that are more representative of human AD pathology.

Another, largely ignored shortcoming of most animal models of AD are sex differences. Exhaustive animal and human data indicate that there are sex differences in cognition, particularly spatial learning (for review see, Li and Singh, 2014). Not surprisingly, there is also a sex difference in AD, with women more likely to develop AD and in some cases have more severe pathology/cognitive impairments (Zhang et al., 1990; Proust-Lima et al., 2008; Viña and Lloret, 2010; for review see, Li and Singh, 2014). While some transgenic models of AD have manipulated sex hormones and assessed cognition/pathology, very few studies have looked at sex differences in their AD model (for review see, Li and Singh, 2014). We predict that it is very likely that males and females in animal models of AD would be differentially affected by the various manipulations used to induce AD pathology. For example, female ApoE4 knockin mice were more impaired in spatial learning tasks and had more severe hippocampal pathology (hilar GABAergic interneurons) than their male counterparts (Leung et al., 2012). It would be interesting to determine if some manipulations used to induce AD are more prone to sex differences than others. Similar to the argument for using episodic memory task and quantifying pathology in the dorsal and ventral hippocampal regions, it is likely that the evaluation of sex differences in animal models of AD would help identify those models that are more akin to human AD.

## Conclusions

Understanding the etiology of the sporadic version of AD, let alone developing cogent strategies for preventing and/or treating this brutal neurodenerative disorder represents an extremely difficult and complex task. In the present paper, we presented just a few of the barriers to the development of a valid rodent model of SAD. These barriers include the use of mutant mouse models of the familial version of this disorder that do not represent the disease state in humans with this rare affliction let alone the masses of aged patients with SAD. A second barrier is that the behavioral tasks predominantly used do not detect partial and heterogenous damage to the hippocampal system. As rodent models of SAD improve, we believe that partial hippocampal dysfunction will be a hallmark, which is consistent with the human clinical literature. Similarly, other characteristics of human SAD, such as sex differences, and mechanism heterogeneity should be considered when developing rodent models of SAD. However, this means that animal researchers will need the behavioral tools for detecting this incomplete damage that will often manifest itself heterogeneously across a large cohort of subjects. If progress on these issues can be made, the ability to accurately model SAD in rodents will be possible and this will allow a better evaluation of neuroprotection and rehabilitation strategies at different developmental time-points for this neurodegenerative disease.

### Conflict of interest statement

The authors declare that the research was conducted in the absence of any commercial or financial relationships that could be construed as a potential conflict of interest.

## References

[B1] Alaverdashvili M. Moon S. K. Beckman C. D. Virag A. Whishaw I. Q. (2008). Acute but not chronic differences in skilled reaching for food following motor cortex devascularization vs. photothrombotic stroke in the rat. Neuroscience 157, 297–308. 10.1016/j.neuroscience.2008.09.015 18848605

[B2] Alz.org. (2014, January 1) Alzheimer's Facts and Figures. Retrieved January 1, 2015, from http://www.alz.org/alzheimers_disease_facts_and_figures.asp#cost.

[B3] Amaral D. G. Lavenex P. (2007). Hippocampal neuroanatomy, in The Hippocampus Book, eds Andersen P. Morris R. Amaral D. Bliss T. O'Keefe J. (New York, NY: Oxford University Press, Inc), 37–114.

[B4] Amaral D. G. Witter M. P. (1989). The three dimensional organization of the hippocampal formation: a review of anatomical data. Neuroscience 31, 571–591. 10.1016/0306-4522(89)90424-7 2687721

[B5] Amtul Z. Nikolova S. Gao L. Keeley J. Bechberger A. L. Fisher R.. (2014a). Co-morbid Aβ toxicity and stroke: hippocampal atrophy, pathology and cognitive deficit. Neurobiol. Aging 35, 1605–1614. 10.1016/j.neurobiolaging.2014.01.005 24491422

[B6] Amtul Z. Whitehead S. N. Keeley R. J. Bechberger J. Fisher A. L. McDonald R. J.. (2014b). Co-morbid rat model of ischemia and β-amyloid toxicity: Striatal and cortical degeneration. Brain Pathol. 25, 24–32. 10.1111/bpa.12149 24725245 PMC8029334

[B7] Andrews J. S. Jansen J. H. Linders S. Princen A. Broekkamp C. L. (1995). Performance of four different rat strains in the autoshaping, two-object discrimination, and swim maze tests of learning and memory. Physiol. Behav. 57, 785–790. 10.1016/0031-9384(94)00336-X 7777618

[B8] Antoniadis E. A. McDonald R. J. (2001). Amygdala, hippocampus, and unconditioned fear. Exp. Brain Res. 138, 200–209. 10.1007/s002210000645 11417461

[B9] Antoniadis E. McDonald R. (2000). Amygdala, hippocampus and discriminative fear conditioning to context. Behav. Brain Res. 108, 25–37. 10.1016/S0166-4328(00)00145-5 10680753

[B10] Antoniadis E. A. McDonald R. J. (1999). Discriminative fear conditioning to context expressed by multiple measures of fear in the rat. Behav. Brain Res. 101, 1–13. 10.1016/S0166-4328(98)00056-4 10342394

[B11] Balducci C. Forloni G. (2011). APP transgenic mice: their use and limitations. Neuromol. Med. 13, 117–137. 10.1007/s12017-010-8141-7 21152995

[B12] Baxter M. G. Bucci D. J. Sobel T. J. Williams M. J. Gorman L. K. Gallagher M. (1996). Intact spatial learning following lesions of basal forebrain cholinergic neurons. Neuroreport 7, 1417–1420. 10.1097/00001756-199605310-00019 8856689

[B13] Berger-Sweeney J. Heckers S. Mesulam M. M. Wiley R. G. Lappi D. A. Sharma M. (1994). Differential effects on spatial navigation of immunotoxin-induced cholinergic lesions of the medial septal area and nucleus basalis magnocellularis. J. Neurosci. 14, 4507–4519. 8027790 10.1523/JNEUROSCI.14-07-04507.1994 PMC6577026

[B14] Braak H. Thal D. R. Ghebremedhin E. Del Tredici K. (2011). Stages of the pathological process in Alzheimer disease: age categories from 1 to 100 years. J. Neuropathol. Exp. Neurol. 70, 960–969. 10.1097/NEN.0b013e318232a379 22002422

[B15] Braak H. Braak E. (1991). Neuropathological stageing of Alzheimer-related changes. Acta Neuropathol. 82, 239–259. 10.1007/BF00308809 1759558

[B16] Braidy N. Muñoz P. Palacios A. G. Castellano-Gonzalez G. Inestrosa N. C. Chung R. S.. (2012). Recent models for Alzheimer's disease: clinical implications and basic research. J. Neural Transm. 119, 173–195. 10.1007/s00702-011-0731-5 22086139

[B17] Borchelt D. R. (1998). Inherited neurodegenerative diseases and transgenic models. Lab. Anim. Sci. 48, 604–610. 10090084

[B18] Borchelt D. R. Ratovitski T. van Lare J. Lee M. K. Gonzales V. Jenkins N. A.. (1997). Accelerated amyloid deposition in the brains of transgenic mice coexpressing mutant presenilin 1 and amyloid precursor proteins. Neuron 19, 939–945. 10.1016/s0896-6273(00)80974-5 9354339

[B19] Brown R. E. (2007). Behavioural phenotyping of transgenic mice. Can. J. Exp. Psychol. 61, 328–344. 10.1037/cjep2007033 18266509

[B20] Cain S. W. Ko C. H. Chalmers J. A. Ralph M. R. (2004). Time of day modulation of conditioned place preference in rats depends on the strain of rat used. Neurobiol. Learn. Mem. 81, 217–220. 10.1016/j.nlm.2004.02.003 15082023

[B21] Choi B. R. Lee S. R. Han J. S. Woo S. K. Kim K. M. Choi D. H.. (2011). Synergistic memory impairment through the interaction of chronic cerebral hypoperfusion and amlyloid toxicity in a rat model. Stroke 42, 2595–2604. 10.1161/STROKEAHA.111.620179 21737797

[B22] Coogan A. N. Schutová B. Husung S. Furczyk K. Baune B. T. Kropp P.. (2013). The circadian system in Alzheimer's disease: disturbances, mechanisms, and opportunities. Biol. Psychiatry 74, 333–339. 10.1016/j.biopsych.2012.11.021 23273723

[B23] Corder E. H. Saunders A. M. Strittmatter W. J. Schmechel D. E. Gaskell P. C. Small G. W.. (1993). Gene dose of apolipoprotein E type 4 allele and the risk of Alzheimer's disease in late onset families. Science 261, 921–923. 10.1126/science.8346443 8346443

[B24] Craig L. A. Hong N. S. Kopp J. McDonald R. J. (2008a). Emergence of spatial impairment in rats following specific cholinergic depletion of the medial septum combined with chronic stress. Eur. J. Neurosci. 27, 2262–2271. 10.1111/j.1460-9568.2008.06179.x 18445217

[B25] Craig L. A. Hong N. S. Kopp J. McDonald R. J. (2008b). Reduced cholinergic status in hippocampus produces spatial memory deficits when combined with kainic acid induced seizures. Hippocampus 18, 1112–1121. 10.1002/hipo.20471 18651618

[B26] Craig L. A. Hong N. S. Kopp J. McDonald R. J. (2009a). Selective lesion of medial septal cholinergic neurons followed by a mini-stroke impairs spatial learning in rats. Exp. Brain Res. 193, 29–42. 10.1007/s00221-008-1592-5 18936927

[B27] Craig L. A. Hong N. S. Kopp J. McDonald R. J. (2009b). Cholinergic depletion of the medial septum followed by phase shifting does not impair memory or rest-activity rhythms measured under standard light/dark conditions in rats. Brain Res. Bull. 79, 53–62. 10.1016/j.brainresbull.2008.10.013 19038315

[B28] Craig L. A. Hong N. S. McDonald R. J. (2011). Revisiting the cholinergic hypothesis in the development of Alzheimer's disease. Neurosci. Biobehav. Rev. 35, 1397–1409. 10.1016/j.neubiorev.2011.03.001 21392524

[B29] Crystal J. D. (2013). Remembering the past and planning for the future in rats. Behav. Process. 93, 39–49. 10.1016/j.beproc.2012.11.014 23219951 PMC3582767

[B30] Crystal J. D. Glanzman D. L. (2013). A biological perspective on memory. Curr. Biol. 23, R728. 10.1016/j.cub.2013.07.082 24028954 PMC5142816

[B31] Czerniawski J. Guzowski J. F. (2014). Acute neuroinflammation impairs context discrimination memory and disrupts pattern separation processes in hippocampus. J. Neurosci. 34, 12470–12480. 10.1523/JNEUROSCI.0542-14.2014 25209285 PMC4160778

[B32] Czerniawski J. Miyashita T. Lewandowski G. Guzowski J. F. (2015). Systemic lipopolysaccharide administration impairs retrieval of context-object discrimination, but not spatial, memory: evidence for selective disruption of specific hippocampus-dependent memory functions during acute neuroinflammation. Brain Behav. Immun. 44, 159–166. 10.1016/j.bbi.2014.09.014 25451612 PMC4358899

[B33] Davies P. Maloney A. J. (1976). Selective loss of central cholinergic neurons in Alzheimer's disease. Lancet 8000, 1403 10.1016/S0140-6736(76)91936-X 63862

[B34] Davis K. E. Easton A. Eacott M. J. Gigg J. (2013a). Episodic-like memory for what-where-which occasion is selectively impaired in the 3xTgAD mouse model of Alzheimer's disease. J. Alzheimers Dis. 33, 681–698. 10.3233/JAD-2012-121543 23034524

[B35] Davis K. E. Eacott M. J. Easton A. Gigg J. (2013b). Episodic-like memory is sensitive to both alzheimer's-like pathological accumulation and normal ageing processes in mice. Beahav. Brain Res. 254, 73–82. 10.1016/j.bbr.2013.03.009 23500896

[B36] de la Torre J. C. (2012). A turning point for Alzheimer's disease. Biofactors 38, 78–83. 10.1002/biof.200 22422426

[B37] Derdikman D. Moser E. I. (2010). A manifold of spatial maps in the brain. Trends Cogn. Sci. 14, 561–569. 10.1016/j.tics.2010.09.004 20951631

[B38] Devan B. D. Goad E. H. Petri H. L. Antoniadis E. A. Hong N. S. Ko C. H.. (2001). Circadian phase-shifted rats show normal acquisition but impaired long-term retention of place information in the water task. Neurobiol. Learn. Mem. 75, 51–62. 10.1006/nlme.1999.3957 11124046

[B39] Dornan W. A. Kang D. E. McCampbell A. Kang E. E. (1993). Bilateral injections of βA(25-35)+IBO into the hippocampus disrupts acquisition of spatial learning in the rat. Neuroreport 5, 165–168. 10.1097/00001756-199311180-00018 8111004

[B40] Driscoll I. Davatzikos C. An Y. Wu X. Shen D. Kraut M. (2009). Longitudinal pattern of regional brain volume change differentiates normal aging from MCI. Neurology 72, 1907–1913. 10.1212/WNL.0b013e3181a82634 PMC2690968 19487648

[B41] Driscoll I. Hong N. S. Craig L. A. Sutherland R. J. McDonald R. J. (2008). Enhanced cell death and learning deficits after a mini-stroke in aged hippocampus. Neurobiol. Aging 29, 1847–1858. 10.1016/j.neurobiolaging.2007.04.025 17561312

[B42] Easton A. Eacott M. J. (2013). Cholinergic mechanisms of episodic memory: what specific behavioural tasks can tell us about specific neural mechanisms. Brain Res. Bull. 92, 21–28. 10.1016/j.brainresbull.2011.09.008 21968024

[B43] Etienne A. S. Maurer R. Saucy F. (1988). Limitations in the assessment of path dependent information. Behaviour 106, 81–111. 10.1163/156853988X00106

[B44] Ferbinteanu J. Holsinger R. M. D. McDonald R. J. (1999). Lesions of the medial or lateral perforant path have different effects on hippocampal contributions to place learning and fear conditioning to context. Behav. Brain Res. 101, 65–84. 10.1016/S0166-4328(98)00144-2 10342401

[B45] Ferbinteanu J. McDonald R. J. (2000). Dorsal and ventral hippocampus: Same or different? Psychobiology 28, 314–324.

[B46] Ferbinteanu J. McDonald R. J. (2001). Dorsal/ventral hippocampus, fornix, and conditioned place preference. Hippocampus 11, 187–200. 10.1002/hipo.1036 11345125

[B47] Ferbinteanu J. McDonald R. J. (2003). Dorsal/ventral hippocampus and spatial learning. Neurosci. Lett. 345, 131–135. 10.1016/S0304-3940(03)00473-7 12821188

[B48] Ferri C. P. Prince M. Brayne C. Brodaty H. Fratiglioni L. Ganguli M.. (2005). Global prevalence of dementia: a Delphi consensus study. Lancet 366, 2112–2117. 10.1016/S0140-6736(05)67889-0 16360788 PMC2850264

[B49] Fjell A. M. McEvoy L. Holland D. Dale A. M. Walhovd K. B. (2014). What is normal in aging? Effects of aging, amyloid and Alzheimer's disease on the cerebral cortex and the hippocampus. Prog. Neurobiol. 117, 20–40. 10.1016/j.pneurobio.2014.02.004 24548606 PMC4343307

[B50] Frank S. Clavaguera F. Tolnay M. (2008). Tauopathy models and human neuropathology: similarities and differences. Acta Neuropathol. 115, 39–53. 10.1007/s00401-007-0291-9 17786456

[B51] Frankland P. W. Cestari V. Filipkowski R. K. McDonald R. J. Silva A. J. (1998). The dorsal hippocampus is essential for context discriminations, but not for context recognition. Behav. Neurosci. 112, 863–874. 10.1037/0735-7044.112.4.863 9733192

[B52] Fuster-Matanzo A. Llorens-Martín M. de Barreda E. G. Ávila J. Hernández F. (2011). Different susceptibility to neurodegeneration of dorsal and ventral hippocampal dentate gyrus: a study with transgenic mice overexpressing GSK3beta. PLoS ONE 6:e27262. 10.1371/journal.pone.0027262 22073301 PMC3207840

[B53] Giannakopoulos P. Herrmann F. R. Bussiere T. Bouras C. Kovari E. Perl D. P.. (2003). Tangle and neuron numbers, but not amyloid load, predict cognitive status in Alzheimer's disease. Neurology 60, 1495–1500. 10.1212/01.WNL.0000063311.58879.01 12743238

[B54] Gil-Bea F. J. Gerenu G. Aisa B. Kirazov L. P. Schliebs R. Ramírez M. J. (2012). Cholinergic denervation exacerbates amyloid pathology and induces hippocampal atrophy in Tg2576 mice. Neurobiol. Dis. 48, 439–446. 10.1016/j.nbd.2012.06.020 22759926

[B55] Grober E. Hall C. B. Lipton R. B. Zonderman A. B. Resnick S. M. Kawas C. (2008). Memory impairment, executive dysfunction, and intellectual decline in preclinical Alzheimer's disease. J. Int. Neuropsychol. Soc. 14, 266–278. 10.1017/s1355617708080302 18282324 PMC2763488

[B56] Gruber A. J. McDonald R. J. (2012). Context, emotion, and the strategic pursuit of goals: interactions among multiple brain systems controlling motivated behavior. Front. Behav. Neurosci. 6:50 10.3389/fnbeh.2012.00050 PMC3411069 22876225

[B57] Hånell A. Marklund N. (2014). Structured evaluation of rodent behavioral tests used in drug discovery research. Front. Behav. Neurosci. 8:252. 10.3389/fnbeh.2014.00252 25100962 PMC4106406

[B58] Hardy J. Selkoe D. J. (2002). The amyloid hypothesis of Alzheimer's disease: progress and problems on the road to therapeutics. Science 297, 353–356. 10.1126/science.1072994 12130773

[B59] Hodges J. R. Patterson K. (1995). Is semantic memory consistently impaired early in the course of Alzheimer's disease? Neuroanatomical and diagnostic implications. Neuropsychologia 33, 441–459. 10.1016/0028-3932(94)00127-B 7617154

[B60] Honea R. A. Cruchaga C. Perea R. D. Saykin A. J. Burns J. M. Weinberger D. R.. (2013). Characterizing the role of brain derived neurotrophic factor genetic variation in Alzheimer's disease neurodegeneration. PLoS ONE 8:e76001. 10.1371/journal.pone.0076001 24086677 PMC3784423

[B61] Huang H. J. Liang K. C. Chang Y. Y. Ke H. C. Lin J. Y. Hsieh-Li H. M. (2010). The interaction between acute oligomer Aβ 1–40 and stress severely impaired spatial learning and memory. Neurobiol. Learn. Mem. 93, 8–18. 10.1016/j.nlm.2009.07.010 19660564

[B62] Hunsaker M. R. Kesner R. P. (2013). The operation of pattern separation and pattern completion processes associated with different attributes or domains of memory. Neurosci. Biobehav. Rev. 37, 36–58. 10.1016/j.neubiorev.2012.09.014 23043857

[B63] Iqbal K. Bolognin S. Wang X. Basurto-Islas G. Blanchard J. Chyn Tnag Y. (2014). Animal models of the sporadic form of Alzheimer's disease. Focus on the disease and not just the lesions. J. Alzheimers Dis. 37, 469–474. 10.3233/JAD-130827 23948903

[B64] Iwasaki K. Egashira N. Hatip-Al-Khatib I. Akiyoshi Y. Arai T. Takagaki Y.. (2006). Cerebral ischemia combined with beta-amyloid impairs spatial memory in the eight-arm radial maze task in rats. Brain Res. 1097, 216–223. 10.1016/j.brainres.2006.04.073 16729978

[B65] Jack C. R. Jr. Knopman D. S. Jagust W. J. Petersen R. C. Weiner M. W. Aisen P. S.. (2013). Tracking pathophysiological processes in Alzheimer's disease: an updated hypothetical model of dynamic biomarkers. Lancet Neurol. 12, 207–216. 10.1016/S1474-4422(12)70291-0 23332364 PMC3622225

[B66] Jack C. R. Knopman D. S. Jagust W. J. Shaw L. M. Aisen P. S. Weiner M. W.. (2010). Hypothetical model of dynamic biomarkers of the Alzheimer's pathological cascade. Lancet Neurol. 9, 119–128. 10.1016/S1474-4422(09)70299-6 20083042 PMC2819840

[B67] Jaspers R. M. A. Block F. Heim C. Sontag K. H. (1990). Spatial learning is affected by transient occlusion of common carotid arteries (2VO): comparison of behavioural and histopathological changes after ‘2VO’ and ‘four-vessel- occlusion’ in rats. Neurosci. Lett. 117, 149–153. 10.1016/0304-3940(90)90135-V 2290611

[B68] Jayatissa M. N. Bisgaard C. F. West M. J. Wiborg O. (2008). The number of granule cells in rat hippocampus is reduced after chronic mild stress and re-established after chronic escitalopram treatment. Neuropharmacology 54, 530–541. 10.1016/j.neuropharm.2007.11.009 18164735

[B69] Ji J. Maren S. (2008). Differential roles for hippocampal areas CA1 and CA3 in the contextual encoding and retrieval of extinguished fear. Learn. Mem. 15, 244–251. 10.1101/lm.794808 18391185 PMC2327266

[B70] Jouvenceau A. Billard J.-M. Lamour Y. Dutar P. (1997). Potentiation of glutamatergic EPSPs in rat CA1 hippocampal neurons after selective cholinergic denervation by 192 IgG-Saporin. Synapse 26, 292–300. 9183818 10.1002/(SICI)1098-2396(199707)26:3<292::AID-SYN10>3.0.CO;2-Y

[B71] Joseph J. Shukitt-Hale B. Denisova N. A. Martin A. Perry G. Smith M. A. (2001). Copernicus revisted: amyloid beta in Alzheimer's disease. Neurobiol. Aging 22, 131–146. 10.1016/S0197-4580(00)00211-6 11164287

[B72] Kalaria R. N. (2000). The role of cerebral ischemia in Alzheimer's disease. Neurobiol. Aging 21, 321–330. 10.1016/S0197-4580(00)00125-1 10867217

[B73] Kawas C. Gray S. Brookmeyer R. Fozard J. Zonderman A. (2000). Age-specific incidence rates of Alzheimer's disease: the Baltimore Longitudinal Study of Aging. Neurology 54, 2072–2077. 10.1212/WNL.54.11.2072 10851365

[B74] Keeley R. J. Hong N. S. Fisher A. McDonald R. J. (2015). Co-morbid beta-amyloid toxicity and stroke produce impairments in an ambiguous context task without any impairments in spatial memory. Neurobiol. Learn. Mem. 119C, 42–51. 10.1016/j.nlm.2015.01.001 25576791

[B75] Kesner R. P. Rolls E. T. (2015). A computational theory of hippocampal function, and tests of the theory: New developments. Neurosci. Biobehav. Rev. 48, 92–147. 10.1016/j.neubiorev.2014.11.009 25446947

[B76] Kim J. Basak J. M. Holtzman D. M. (2009). The role of apolopoprotein E in Alzheimer's disease. Neuron 63, 287–303. 10.1016/j.neuron.2009.06.026 19679070 PMC3044446

[B77] Kim J. J. Fanselow M. S. (1992). Modality-specific retrograde amnesia of fear. Science 256, 675–677. 10.1126/science.1585183 1585183

[B78] Kimmelman J. London A. J. (2011). Predicting harms and benefits in translational trials: ethics, evidence, and uncertainty. PLoS Med. 8:e1001010. 10.1371/journal.pmed.1001010 21423344 PMC3050916

[B79] Klunk W. E. Engler H. Nordberg A. Wang Y. Blomqvist G. Holt D. P.. (2004). Imaging brain amyloid in Alzheimer's disease with Pittsburgh Compound−B. Ann. Neurol. 55, 306–319. 10.1002/ana.20009 14991808

[B80] Knierim J. J. Neunuebel J. P. Deshmukh S. S. (2014). Functional correlates of the lateral and medial entorhinal cortex: objects, path integration and local-global reference frames. Philos. Trans. R. Soc. Lond. B Biol. Sci. 369:20130369. 10.1098/rstb.2013.0369 24366146 PMC3866456

[B81] La Joie R. Perrotin A. de La Sayette V. Egret S. Doeuvre L. Belliard S.. (2013). Hippocampal subfield volumetry in mild cognitive impairment, Alzheimer's disease and semantic dementia. Neuroimage Clin. 3, 155–162. 10.1016/j.nicl.2013.08.007 24179859 PMC3791274

[B82] Lazarov O. Marr R. A. (2013). Of mice and men: neurogenesis, cognition and Alzheimer's disease. Front. Aging Neurosci. 5:43. 10.3389/fnagi.2013.00043 23986699 PMC3753540

[B83] Lecanu L. Papadopoulos V. (2013). Modeling Alzheimer's disease with non-transgenic rat models. Alzheimers Res. Ther. 5, 1–9. 10.1186/alzrt171 23634826 PMC3706888

[B84] Lehmann O. Grottick A. J. Cassel J.-C. Higgins G. A. (2003). A double dissociation between serial reaction time and radial maze performance in rats subjected to 192 IgG-saporin lesions of the nucleus basalis and/or the septal region. Eur. J. Neurosci. 18, 651–666. 10.1046/j.1460-9568.2003.02745.x 12911761

[B85] Lehmann H. Lacanilao S. Sutherland R. J. (2007). Complete or partial hippocampal damage produces equivalent retrograde amnesia for remote contextual fear memories. Eur. J. Neurosci. 25, 1278–1286. 10.1111/j.1460-9568.2007.05374.x 17355254

[B86] Lehmann H. Sparks F. T. Spanswick S. C. Hadikin C. McDonald R. J. Sutherland R. J. (2009). Making context memories independent of the hippocampus. Learn. Mem. 16, 417–420. 10.1101/lm.1385409 19553378 PMC2704104

[B87] Lescaudron L. Verna A. (1985). Effects of chronic ethanol consumption on pyramidal neurons of the mouse dorsal and ventral hippocampus: a quantitative histological analysis. Exp. Brain Res. 58, 362–367. 10.1007/BF00235317 4039674

[B88] Leung L. Andrews-Zwilling Y. Yoon S. Y. Jain S. Ring K. Dai J.. (2012). Apolipoprotein E4 causes age- and sex- dependent impairments of hilar GABAergic interneurons and learning and memory deficits in mice. PLoS ONE 7:e53569. 10.1371/journal.pone.0053569 23300939 PMC3534053

[B89] Leutgeb J. K. Leutgeb S. Moser M-B. Moser E. I. (2007). Pattern separation in the dentate gyrus and CA3 of the hippocampus. Science 315, 961–966. 10.1126/science.1135801 17303747

[B90] Leutgeb S. Leutgeb J. K. Moser M. B. Moser E. I. (2005). Place cells, spatial maps and the population code for memory. Curr. Opin. Neurobiol. 15, 738–746. 10.1016/j.conb.2005.10.002 16263261

[B91] Leutgeb S. Leutgeb J. K. Treves A. Moser M. B. Moser E. I. (2004). Distinct ensemble codes in hippocampal areas CA3 and CA1. Science 305, 1295–1298. 10.1126/science.1100265 15272123

[B92] Lewis J. Dickson D. W. Lin W. L. Chisholm L. Corral A. Jones G.. (2001). Enhanced neurofibrillary degeneration in transgenic mice expressing mutant tau and APP. Science 293, 1487–1491. 10.1126/science.1058189 11520987

[B93] Li R. Lindholm K. Yang L-B. Yue X. Citron M. Yan R.. (2004). Amyloid β peptide load is correlated with increased β-secretase activity in sporadic Alzheimer's disease patients. Proc. Natl. Acad. Sci. U.S.A. 101, 3632–3637. 10.1073/pnas.0205689101 14978286 PMC373514

[B94] Li R. Singh M. (2014). Sex differences in cognitive impairment and Alzheimer's disease. Front. Neuroendocrinol. 35, 385–403. 10.1016/j.yfrne.2014.01.002 24434111 PMC4087048

[B95] Lupien S. J. de Leon M. de Santi S. Convit A. Tarshish C. Nair N. P. V.. (1998). Cortisol levels during human aging predict hippocampal atrophy and memory deficits. Nat. Neurosci. 1, 69–73. 10.1038/271 10195112

[B96] Maaswinkel H. Jarrard L. E. Whishaw I. Q. (1999). Hippocampectomized rats are impaired in homing by path integration. Hippocampus 9, 53–561. 10560926 10.1002/(SICI)1098-1063(1999)9:5<553::AID-HIPO9>3.0.CO;2-G

[B97] Madrigal J. L. M. Caso J. R. Cristobal J. D. Cardenas A. Leza J. C. Lizoasoain I.. (2003). Effect of subacute and chronic immobilization stress on the outcome of permanent focal cerebral ischemia in rats. Brain Res. 979, 137–135. 10.1016/S0006-8993(03)02892-0 12850580

[B98] Mahley R. W. Rall S. C. Jr. (2000). Apolipoprotein E: far more than a lipid transport protein. Annu. Rev. Genomics Hum. Genet. 1, 507–537. 10.1146/annurev.genom.1.1.507 11701639

[B99] Mankin E. A. Sparks F. T. Slayyeh B. Sutherland R. J. Leutgeb S. Leutgeb J. K. (2012). Neuronal code for extended time in the hippocampus. Proc. Natl. Acad. Sci.U.S.A 109, 19462–19467. 10.1073/pnas.1214107109 23132944 PMC3511087

[B100] Maren S. Aharonov G. Fanselow M. (1997). Neurotoxic lesions of the dorsal hippocampus and pavlovian fear conditioning in rats. Behav. Brain Res. 88, 261–274. 10.1016/S0166-4328(97)00088-0 9404635

[B101] Martin M. M. Wallace D. G. (2007). Selective hippocampal cholinergic deafferenation impairs self-movement cue use during a food hoarding task. Behav. Brain Res. 183, 78–86. 10.1016/j.bbr.2007.05.026 17610963 PMC1987711

[B102] Maruszak A. Thuret S. (2014). Why looking at the whole hippocampus is not enough – a critical role for anteroposterior axis, subfield and activation analyses to enhance predictive value of hippocampal changes for Alzheimer's disease diagnosis. Front. Cell. Neurosci. 8:95 10.3389/fncel.2014.00095 PMC3978283 24744700

[B103] Maruszak A. Pilarski A. Murphy T. Branch N. Thuret S. (2013). Hippocampal neurogenesis in Alzheimer's disease: is there a role for dietary modulation? J. Alzheimers Dis. 38, 11–38. 10.3233/JAD-131004 23948932

[B104] McClelland J. L. McNaughton B. L. O'Reilly R. C. (1995). Why are there complimentary learning systems in the hippocampus and neocortex: insights from the successes and failures of connectionist models of learning and memory. Psychol. Rev. 102, 419–457. 10.1037/0033-295X.102.3.419 7624455

[B105] McDonald R. J. (2002). Multiple co-factors produce variants of age-related cognitive decline: a theory. Can. J. Exp. Psychol. 56, 221–239. 10.1037/h0087399 12271752

[B106] McDonald R. J. Craig L. A. Hong N. S. (2008). Enhanced cell death in the hippocampus and emergence of cognitive impairment following a localized mini-stroke in hippocampus if preceded by a previous episode of acute stress. Eur. J. Neurosci. 27, 2197–2209. 10.1111/j.1460-9568.2008.06151.x 18412637

[B107] McDonald R. J. Craig L. A. Hong N. S. (2010). The etiology of age-related dementia is more complicated than we think. Behav. Brain Res. 214, 3–11. 10.1016/j.bbr.2010.05.005 20471430

[B108] McDonald R. J. Hong N. S. Devan B. D. (2004). The challenges of understanding mammalian cognition and memory-based behaviours: an interacting learning and memory systems approach. Neurosci. Biobehav. Rev. 28, 719–746. 10.1016/j.neubiorev.2004.09.014 15555681

[B109] McDonald R. J. Murphy R. A. Guarraci F. A. Gortler J. R. White N. M. Baker A. G. (1997). A systematic comparison of the effects of hippocampal and fornix-fimbria lesions on acquisition of three configural discrimination tasks. Hippocampus 7, 371–388. 9287077 10.1002/(SICI)1098-1063(1997)7:4<371::AID-HIPO3>3.0.CO;2-M

[B110] McDonald R. J. White N. M. (1994). Parallel information processing in the water maze: evidence for independent memory systems involving dorsal striatum and hippocampus. Behav. Neural Biol. 61, 260–270. 10.1016/S0163-1047(05)80009-3 8067981

[B111] McDonald R. J. White N. M. (1995a). Hippocampal and non-hippocampal contributions to place learning. Behav. Neurosci. 109, 579–593. 10.1037/0735-7044.109.4.579 7576202

[B112] McDonald R. J. White N. M. (1995b). Information acquired by the hippocampus interferes with acquisition of the amygdala-based conditioned-cue preference in the rat. Hippocampus 5, 189–197. 10.1002/hipo.450050305 7550614

[B113] Maguire E. (2001). The retrosplenial contribution to human navigation: a review of lesion and neuroimaging findings. Scand. J. Psychol. 42, 225–238. 10.1111/1467-9450.00233 11501737

[B114] McNaughton B. L. Barnes C. A. O'Keefe J. (1983). The contributions of position, direction, and velocity to single unit activity in the hippocampus of freely-moving rats. Exp. Brain Res. 52, 41–49. 10.1007/BF00237147 6628596

[B115] Mesulam M. (2004). The cholinergic lesion of Alzheimer's disease: pivotal factor or side show? Learn. Mem. 11, 43–49. 10.1101/lm.69204 14747516

[B116] Metz G. A. Antonow-Schlorke I. Witte O. W. (2005). Motor improvements after focal cortical ischemia in adult rats are mediated by compensatory mechanisms. Behav. Brain Res. 162, 71–82. 10.1016/j.bbr.2005.03.002 15922067

[B117] Mormino E. C. (2014). The relevance of Beta-amyloid on marekers of Alzheimer's disease in clinically normal individuals and factors that influence these associations. Neuropsychol. Rev. 24, 300–312. 10.1007/s11065-014-9267-4 25108368 PMC4313873

[B118] Moser E. I. Kropff E. Moser M. B. (2008). Place cells, grid cells, and the brain's spatial representation system. Annu. Rev. Neurosci. 31, 69–89. 10.1146/annurev.neuro.31.061307.090723 18284371

[B119] Moser M. B. Moser E. I. (1998). Distributed encoding and retrieval of spatial memory in the hippocampus. J. Neurosci. 18, 7535–7542. 9736671 10.1523/JNEUROSCI.18-18-07535.1998 PMC6793256

[B120] Moser E. Moser M. B. Andersen P. (1993). Spatial learning impairment parallels the magnitude of dorsal hippocampal lesions, but is hardly present following ventral lesions. J. Neurosci. 13, 3916–3925. 8366351 10.1523/JNEUROSCI.13-09-03916.1993 PMC6576447

[B121] Moser M. B. Moser E. I. Forrest E. Andersen P. Morris R. G. (1995). Spatial learning with a minislab in the dorsal hippocampus. Proc. Natl. Acad. Sci.U.S.A. 92, 9697–9701. 10.1073/pnas.92.21.9697 7568200 PMC40869

[B122] Morris R. G. Garrud P. Rawlins J. N. O'Keefe J. (1982). Place navigation impaired in rats with hippocampal lesions. Nature 297, 681–683. 10.1038/297681a0 7088155

[B123] Morris R. G. M. (2007). Theories of hippocampal function, in The Hippocampus Book, eds Andersen P. Morris R. Amaral D. Bliss T. O'Keefe J. (New York, NY: Oxford University Press), 581–694.

[B124] Muller R. U. Kubie J. L. (1987). The effects of changes in the environment on the spatial firing of hippocampal complex-spike cells. J. Neurosci. 7, 1951–1968 3612226 10.1523/JNEUROSCI.07-07-01951.1987 PMC6568940

[B125] Näslund J. Haroutunian V. Mohs R. Davis K. L. Davies P. Greengard P.. (2000). Correlation between elevated levels of amyloid β-peptide in the brain and cognitive decline. JAMA 283, 1571–1577. 10.1001/jama.283.12.1571 10735393

[B126] Oddo S. Caccamo A. Shepherd J. D. Murphy M. P. Golde T. E. Kayed R.. (2003). Triple-transgenic model of Alzheimer's disease with plaques and tangles: intracellular Abeta and synaptic dysfunction. Neuron 39, 409–421. 10.1016/S0896-6273(03)00434-3 12895417

[B127] Okada T. Yamada N. Tsuzuki K. Horikawa H. P. Tanaka K. Ozawa S. (2003). Long-term potentiation in the hippocampal CA1 area and dentate gyrus plays different roles in spatial learning. Eur. J. Neurosci. 17, 341–349. 10.1046/j.1460-9568.2003.02458.x 12542671

[B128] O'Keefe J. Dostrovsky J. (1971). The hippocampus as a spatial map. Preliminary evidence from unit activity in the freely-moving rat. Brain Res. 34, 171–175. 5124915 10.1016/0006-8993(71)90358-1

[B129] O'Keefe J. Nadel L. (1978). The Hippocampus as a Cognitive Map. Oxford: Clarendon Press.

[B130] O'Keefe J. Speakman A. (1987). Single unit activity in the rat hippocampus during a spatial memory task. Exp. Brain Res. 68, 1–27. 10.1007/BF00255230 3691688

[B131] Olton D. S. Becker J. T. Handelmann G. E. (1979). Hippocampus, space and memory. Behav. Brain Sci. 2, 313–365. 10.1017/S0140525X00062713

[B132] Paré W. P. (1996). Enhanced retrieval of unpleasant memories influenced by shock controllability, shock sequence, and rat strain. Biol. Psychiatry 39, 808–813. 10.1016/0006-3223(95)00220-0 8731522

[B133] Perry R. J. Watson P. Hodges J. R. (2000). The nature and staging of attention dysfunction in early (minimal to mild) Alzheimer's disease: relationship to episodic and semantic memory impairment. Neuropsychologia 38, 252–271. 10.1016/S0028-3932(99)00079-2 10678692

[B134] Prince M. Bryce R. Albanese E. Wimo A. Ribeiro W. Ferri C. P. (2013). The global prevalence of dementia: a systematic review and metaanalysis. Alzheimers Dement. 9, 63–7500. 10.1016/j.jalz.2012.11.007 23305823

[B135] Proust-Lima C. Amieva H. Letenneur L. Orgogozo J. M. Jacqmin-Gadda H. Dartigues J. F. (2008). Gender and education impact on brain aging: a general cognitive factor approach. Psychol. Aging 23, 608–620. 10.1037/a0012838 18808250

[B136] Pryce C. R. Lehmann J. Feldon J. (1999). Effect of sex on fear conditioning is similar for context and discrete CS in Wistar, Lewis and Fischer rat strains. Pharmacol. Biochem. Behav. 64, 753–759. 10.1016/S0091-3057(99)00147-1 10593198

[B137] Raji C. A. Lopez O. L. Kuller L. H. Carmichael O. T. Becker J. T. (2009). Age, Alzheimer disease, and brain structure. Neurology 73, 1899–1905. 10.1212/WNL.0b013e3181c3f293 19846828 PMC2788799

[B138] Ramos-Rodriguez J. J. Pacheco-Herrero M. Thyssen D. Murillo-Carretero M. I. Berrocoso E. Spires-Jones T. L.. (2013). Rapid β-amyloid deposition and cognitive impairment after cholinergic denervation in APP/PS1 mice. J. Neuropathol. Exp. Neurol. 72, 272. 10.1097/NEN.0b013e318288a8dd 23481704 PMC3612835

[B139] Rodríguez J. J. Jones V. C. Tabuchi M. Allan S. M. Knight E. M. LaFerla F. M.. (2008). Impaired adult neurogenesis in the dentate gyrus of a triple transgenic mouse model of Alzheimer's disease. PLoS ONE 3:e2935. 10.1371/journal.pone.0002935 18698410 PMC2492828

[B140] Ruediger S. Spirig D. Donato F. Caroni P. (2012). Goal-oriented searching mediated by ventral hippocampus early in trial-and-error learning. Nat. Neurosci. 15, 1563–1573. 10.1038/nn.3224 23001061

[B141] Saito T. Matsuba Y. Mihira N. Takano J. Nilsson P. Itohara S.. (2014). Single *App* knock-in mouse models of Alzheimer'disease. Nat. Neurosci. 17, 661–664. 10.1038/nn.3697 24728269

[B142] Sapolsky R. M. (1999). Glucocorticoids, stress, and their adverse neurological effects: relevance to aging. Exp. Gerontol. 34, 721–732. 10.1016/S0531-5565(99)00047-9 10579633

[B143] Schellenberg G. D. Montine T. J. (2012). The genetics and neuropathology of Alzheimer's disease. Acta Neuropathol. 124, 305–323. 10.1007/s00401-012-0996-2 22618995 PMC3708460

[B144] Schneider J. A. Arvanitakis Z. Bang W. Bennett D. A. (2007). Mixed brain pathologies account for most dementia cases in community-dwelling older persons. Neurology 69, 2197–2204. 10.1212/01.wnl.0000271090.28148.24 17568013

[B145] Selkoe D. J. (2002). Alzheimer's disease is a synaptic failure. Science 298, 789–791. 10.1126/science.1074069 12399581

[B146] Serino S. Cipresso P. Morganti F. Riva G. (2014). The role of egocentric and allocentric abilities in Alzheimer's disease: a systematic review. Ageing Res. Rev. 16, 32–44. 10.1016/j.arr.2014.04.004 24943907

[B147] Silveira D. C. Holmes G. L. Schachter S. C. Geula C. Schomer D. L. (2000). Increased susceptibility to generalized seizures after immunolesions of the basal forebrain cholinergic neurons in rats. Brain Res. 878, 223–227. 10.1016/S0006-8993(00)02703-7 10996157

[B148] Small S. A. (2014). Isolating pathogenic mechanisms embedded within the hippocampal circuit through regional vulnerability. Neuron 84, 32–39. 10.1016/j.neuron.2014.08.030 25277453 PMC4185396

[B149] Snowdon D. A. Greiner L. H. Mortimer J. A. Riley K. P. Greiner P. A. Markesbery W. R. (1997). Brain infarction and the clinical expression of Alzheimer's disease: the nun study. JAMA 277, 813–817. 10.1001/jama.1997.03540340047031 9052711

[B150] Sodhi R. K. Jaggi A. S. Singh N. (2014). Animal models of dementia and cognitive dysfunction. Life Sci. 109, 73–86. 10.1016/j.lfs.2014.05.017 25066372

[B151] Spanswick S. C. Sutherland R. J. (2010). Object/context-specific memory deficits associated with loss of hippocampal granule cells after adrenalectomy in rats. Learn. Mem. 17, 241–245. 10.1101/lm.1746710 20410060 PMC2893217

[B152] Sparks F. T. (2011). Interactions of Hippocampal and Non-hippocampal Long-term Memory Systems during Learning, Remembering, and Over Time. Doctoral dissertation, University of Lethbridge.

[B153] Stewart R. Liolitsa D. (1999). Type 2 diabetes mellitus, cognitive impairment and dementia. Diabet. Med. 16, 93–112. 10.1046/j.1464-5491.1999.00027.x 10229302

[B154] Srinivasan V. Pandi-Perumal S. R. Cardinali D. P. Poeggeler B. Hardeland R. (2006). Melatonin in Alzheimer's disease and other neurodegenerative disorders. Behav. Brain Funct. 2:15. 10.1186/1744-9081-2-15 16674804 PMC1483829

[B155] Sutherland R. J. McDonald R. J. (1990). Hippocampus, amygdala, and memory deficits in rats. Behav. Brain Res. 37, 57–79. 10.1016/0166-4328(90)90072-M 2310495

[B156] Sutherland R. J. McDonald R. J. Savage D. D. (2000). Prenatal exposure to moderate levels of ethanol can have long-lasting effects learning and memory in adult offspring. Psychobiology 28, 532–539. 10.3758/BF03332012 9136052

[B157] Sutherland R. J. Rudy J. W. (1988). Place learning in the Morris place navigation task is impaired by damage to the hippocampal formation even if the temporal demands are reduced. Psychobiology 16, 157–163.

[B158] Sutherland R. J. Rudy J. W. (1989). Configural association theory: the role of the hippocampal formation in learning, memory, and amnesia. Psychobiology 17, 129–144.

[B159] Sutherland R. J. Whishaw I. Q. Kolb B. (1983). A behavioural analysis of spatial localization following electrolytic, kainate- or cochicine-induced damage to the hippocampal formation in the rat. Behav. Brain Res. 7, 133–153. 10.1016/0166-4328(83)90188-2 6830648

[B160] Tackenberg C. Brandt R. (2009). Divergent pathways mediate spine alterations and cell death induced by amyloid-β, wild-type tau, and R406W tau. J. Neurosci. 29, 14439–14450. 10.1523/JNEUROSCI.3590-09.2009 19923278 PMC6665808

[B161] Taube J. S. (1998). Head direction cells and the neurophysiological basis for a sense of direction. Prod. Neurobiol. 55, 225–256. 10.1016/S0301-0082(98)00004-5 9643555

[B162] Thorpe C. M. Deibel S. H. Reddigan J. I. Fontaine C. J. (2012). Strain differences in a high response-cost daily time-place learning task. Behav. Processes 90, 384–391. 10.1016/j.beproc.2012.04.004 22542459

[B163] Tinius T. P. Beckwith B. E. Oltmanns D. (1989). Arginine vasopressin facilitates reversal learning in albino, but not hooded rats. Peptides 10, 237–239. 10.1016/0196-9781(89)90099-5 2748420

[B164] Tong X. K. Nicolakakis N. Kocharyan A. Hamel E. (2005). Vascular remodeling versus beta-amyloid induced oxidative stress in the cerebrovascular dysfunctions associated with Alzheimer's disease. J. Neurosci. 25, 11165–11174. 10.1523/JNEUROSCI.4031-05.2005 16319316 PMC6725645

[B165] Tulving E. Markowitsch H. J. (1998). Episodic and declarative memory: role of the hippocampus. Hippocampus 8, 198–204. 9662134 10.1002/(SICI)1098-1063(1998)8:3<198::AID-HIPO2>3.0.CO;2-G

[B166] Tulving E. (1993). What is episodic memory? Curr. Dir. Psychol. Sci. 2, 67–70. 10.1111/1467-8721.ep10770899

[B167] Umeda T. Maekawa S. Kimura T. Takashima A. Tomiyama T. Mori H. (2014). Neurofibrillary tangle formation by introducing wild-type human tau into APP transgenic mice. Acta Neuropathol. 127, 685–698. 10.1007/s00401-014-1259-1 24531886

[B168] Vann S. D. Aggleton J. P. Maguire E. A. (2009). What does the retrosplenial cortex do? Nat. Rev. Neurosci. 10, 792–802. 10.1038/nrn2733 19812579

[B169] van der Staay F. J. Schuurman T. van Reenen C. G. Korte S. M. (2009). Emotional reactivity and cognitive performance in aversively motivated tasks: a comparison between four rat strains. Behav. Brain Funct. 5:50. 10.1186/1744-9081-5-50 20003525 PMC2805679

[B170] Villemagne V. L. Burnham S. Bourgeat P. Brown B. Ellis K. A. Salvado O.. (2013). Amyloid beta deposition, neurodegeneration, and cognitive decline in sporadic Alzheimer's disease: a prospective cohort study. Lancet Neurol. 12, 357–367. 10.1016/S1474-4422(13)70044-9 23477989

[B171] Viña J. Lloret A. (2010). Why women have more Alzheimer's disease than men: gender and mitochondrial toxicity of amyloid-beta peptide. J. Alzheimers. Dis. 20, S527–S533. 10.3233/JAD-2010-100501 20442496

[B172] Wahl F. Allix M. Plotkine M. Boulu R. G. (1992). Neurological and behavioural outcomes of focal ischemia in rats. Stroke 23, 267–272. 10.1161/01.STR.23.2.267 1561657

[B173] Whishaw I. Q. Tomie J.-A. (1996). Of mice and mazes: similarities between mice and rats on dry land but not water mazes. Physiol. Behav. 60, 1191–1197. 10.1016/S0031-9384(96)00176-X 8916170

[B174] White N. M. McDonald R. J. (2002). Multiple memory systems in the rat brain: a review. Neurobiol. Learn. Mem. 77, 125–184. 10.1006/nlme.2001.4008 11848717

[B175] Whitehead S. N. Hachinksi V. C. Cechetto D. F. (2005a). Interaction between a rat model of Aβ toxicity and cerebral ischemia: I. Inflammatory Responses. Stroke 36, 107–112. 10.1161/01.STR.0000149627.30763.f9 15591213

[B176] Whitehead S. N. Cheng G. V. Hachinksi V. Cechetto D. F. (2005b). Interaction between a rat model of Aβ toxicity and cerebral ischemia: II. Effects of triflusal, a Cox-2 inhibitor. Stroke 36, 1782–1789. 10.1161/01.STR.0000173405.02425.d6 16040593

[B177] Whitehead S. N. Cheng G. Hachinksi V. C. Cechetto D. F. (2007). Progressive increase in infarct size, neuroinflammation and cognitive deficits in the presence of high levels of amyloid. Stroke 38, 3245–3250. 10.1161/STROKEAHA.107.492660 17962591

[B178] Witter M. P. Wouterlood F. G. Naber P. A. van Haeften T. (2000). Anatomical organization of the parahippocampal-hippocampal network. Ann. N. Y. Acad. Sci. 911, 1–24. 10.1111/j.1749-6632.2000.tb06716.x 10911864

[B179] Wilber A. A. Clark B. J. Forster T. C. Tatsuno M. McNaughton B. L. (2014). Interaction of egocentric and world-centered reference frames in the rat posterior parietal cortex. J. Neurosci. 34, 5431–5446. 10.1523/JNEUROSCI.0511-14.2014 24741034 PMC3988403

[B180] Wiltgen B. J. Sanders M. J. Anagnostaras S. G. Sage J. R. Fanselow M. S. (2006). Context fear learning in the absence of the hippocampus. J. Neurosci. 26, 5484–5491. 10.1523/JNEUROSCI.2685-05.2006 16707800 PMC6675287

[B181] Wixted J. T. Squire L. R. Jang Y. Papesh M. H. Goldinger S. D. Kuhn J. R.. (2014). Sparse and distributed coding of episodic memory in neurons of the human hippocampus. Proc. Natl. Acad. Sci. U.S.A. 111, 9621–9626. 10.1073/pnas.1408365111 24979802 PMC4084456

[B182] Yamaguchi T. Suzuki M. Yamamoto M. (1995). YM796, a novel muscarinic agonist, improves the impairment of learning behaviour in a rat model of chronic focal cerebral ischemia. Brain Res. 669, 107–114. 10.1016/0006-8993(94)01268-M 7712153

[B183] Young J. W. Jentsch J. D. Bussey T. J. Wallace T. L. Hutcheson D. M. (2013). Consideration of species differences in developing novel molecules as cognition enhancers. Neurosci. Biobehav. Rev. 37, 2181–2193. 10.1016/j.neubiorev.2012.10.002 23064177 PMC3594426

[B184] Zelinski E. L. Hong N. S. McDonald R. J. (2014). Persistent impairments in hippocampal function following a brief series of photoperiod shifts in rats. Anim. Cogn. 17, 127–141. 10.1007/s10071-013-0645-8 23728615

[B185] Zhang M. Y. Katzman R. Salmon D. Jin H. Cai G. J. Wang Z. Y.. (1990). The prevalence of dementia and Alzheimer's disease in Shanghai, China: impact of age, gender, and education. Ann. Neurol. 27, 428–437. 10.1002/ana.410270412 2353798

[B186] Zhou W. Hohmann A. G. Crystal J. D. (2012). Rats answer an unexpected question after incidental encoding. Curr. Biol. 22, 1149–1153. 10.1016/j.cub.2012.04.040 22633809 PMC3376895

